# Genetic Variability in Oxidative Stress, Inflammatory, and Neurodevelopmental Pathways: Impact on the Susceptibility and Course of Spinal Muscular Atrophy

**DOI:** 10.1007/s10571-024-01508-y

**Published:** 2024-10-27

**Authors:** Maruša Barbo, Blaž Koritnik, Lea Leonardis, Tanja Blagus, Vita Dolžan, Metka Ravnik-Glavač

**Affiliations:** 1 Institute of Biochemistry and Molecular Genetics, Faculty of Medicine, University of Ljubljana, Ljubljana, Slovenia; 2 Institute of Clinical Neurophysiology, University Medical Centre Ljubljana, Ljubljana, Slovenia; 3 Department of Neurology, Faculty of Medicine, University of Ljubljana, Ljubljana, Slovenia; 4 Ljubljana, Slovenia

**Keywords:** Spinal muscular atrophy, Oxidative stress, Inflammation, Neurodevelopment, Single nucleotide polymorphism, Neurodegeneration

## Abstract

The spinal muscular atrophy (SMA) phenotype strongly correlates with the *SMN2* gene copy number. However, the severity and progression of the disease vary widely even among affected individuals with identical copy numbers. This study aimed to investigate the impact of genetic variability in oxidative stress, inflammatory, and neurodevelopmental pathways on SMA susceptibility and clinical progression. Genotyping for 31 genetic variants across 20 genes was conducted in 54 SMA patients and 163 healthy controls. Our results revealed associations between specific polymorphisms and SMA susceptibility, disease type, age at symptom onset, and motor and respiratory function. Notably, the *TNF* rs1800629 and *BDNF* rs6265 polymorphisms demonstrated a protective effect against SMA susceptibility, whereas the *IL6* rs1800795 was associated with an increased risk. The polymorphisms *CARD8* rs2043211 and *BDNF* rs6265 were associated with SMA type, while *SOD2* rs4880, *CAT* rs1001179, and *MIR146A* rs2910164 were associated with age at onset of symptoms after adjustment for clinical parameters. In addition, *GPX1* rs1050450 and *HMOX1* rs2071747 were associated with motor function scores and lung function scores, while *MIR146A* rs2910164, *NOTCH* rs367398 SNPs, and *GSTM1* deletion were associated with motor and upper limb function scores, and *BDNF* rs6265 was associated with lung function scores after adjustment. These findings emphasize the potential of genetic variability in oxidative stress, inflammatory processes, and neurodevelopmental pathways to elucidate the complex course of SMA. Further exploration of these pathways offers a promising avenue for developing personalized therapeutic strategies for SMA patients.

**Supplementary Information:**

The online version contains supplementary material available at 10.1007/s10571-024-01508-y.

## Introduction

Spinal muscular atrophy (SMA) is an autosomal recessive neurodegenerative disease that leads to progressive muscle wasting due to the selective loss of spinal cord motor neurons (MNs) caused by mutations in the survival of motor neuron 1 gene (*SMN1*) (Crawford & Pardo 1996; D'Amico et al. 2011; Lefebvre et al. 1995). This disease leads to progressive loss of motor function and reduced life expectancy (Hamilton & Gillingwater 2013; Lorson et al. 1999). On chromosome 5q13, there are two highly homologous genes in humans, *SMN1* and survival of motor neuron 2 gene (*SMN2*), which encode for the survival of motor neuron protein (SMN). A reduced amount of SMN due to *SMN1* gene deletions and/or point mutations is the cause of SMA in most cases (Frugier et al. 2002). While *SMN1* encodes full-length SMN transcripts*, SMN2* mainly generates SMN transcripts lacking exon 7. This leads to the formation of a truncated, nonfunctional SMN protein from 85 to 90% of the mRNA derived from *SMN2*. A functional full-length SMN protein is produced from the remaining 10–15% of *SMN2*-derived mRNAs but not in sufficient quantity to compensate for the loss of *SMN1* (Kashima & Manley 2003). An important factor in the variation in the phenotype of SMA patients is the variation in *SMN2* copy number between individuals, which is partly responsible for the observed differences in disease severity between affected individuals (D'Amico et al. 2011; Feldkötter et al. 2002). In particular, disease severity is usually inversely proportional to the *SMN2* gene copy number (Burghes 1997).

Because SMA is highly prevalent, it has traditionally been clinically categorized by the International Spinal Muscular Atrophy Consortium into three types based on the age of onset and the greatest motor milestone achieved—from the most severe type 1 to the mildest type 3 (Munsat & Davies 1992). Finally, some experts argue for a broader classification that includes other types, such as congenital SMA (type 0) (Dubowitz 1999) and adult-onset SMA (type 4). In SMA type 4, affected individuals reach motor milestones in childhood but do not develop symptoms until adulthood (Mercuri et al. 2012; Zerres et al. 1995).

The first drug approved for the treatment of SMA was nusinersen (Spinraza®), an antisense oligonucleotide designed to increase the production of the SMN protein. In addition to intrathecally administered nusinersen, the orally administered small molecule *SMN2* splicing modifier risdiplam (Evrysdi®) is approved for the treatment of SMA (Ratni et al. 2018). Alongside abeparvovec (Zolgensma®), an adeno-associated viral vector-based gene therapy, these drugs constitute the main treatments aimed at restoring the levels of SMN protein involved in mRNA splicing (Pellizzoni et al. 1998). Despite the critical role of the SMN protein in maintaining muscle and MN function, which leads to progressive MN loss and skeletal muscle atrophy in individuals with deficiency, the exact underlying mechanisms have yet to be fully elucidated (Ando et al. 2020; Bowerman et al. 2017).

The physiological formation of reactive oxygen species (ROS) is normally balanced by ROS-scavenging enzymes, however, prolonged exposure to high levels of ROS can lead to oxidative stress. Elevated ROS levels observed in both cellular models and postmortem tissue from SMA patients suggest that oxidative stress is an important factor in the pathogenesis of SMA. In particular, abnormal accumulation of 4-hydroxy-2-nonenal, a reactive lipid aldehyde produced by lipid peroxidation, has been found in the brainstem and spine of SMA type 1 patients (Hayashi et al. 2002). While some studies in cellular models of mice and humans showed an overall increase in total or mitochondrial ROS production (Acsadi et al. 2009; Ando et al. 2017; Miller et al. 2016; Thelen et al. 2020; Wang et al. 2013), others reported conflicting results, emphasizing the variability of the model systems and the developmental stages in which the measurements were performed (Patitucci & Ebert 2016). Notably, increased mitochondrial ROS production was found only in human stem cell-derived MNs but not in forebrain neurons (Wang et al. 2013). These findings suggest that mitochondrial oxidative stress plays a role in the degeneration of spinal MNs – a process, which is specific to SMA (Zilio et al. 2022).

Furthermore, oxidative stress in the SMA affects not only the MNs but also the muscles. ROS are physiologically generated by increased mitochondrial respiration during muscle contraction (Barbieri & Sestili 2012). However, elevated ROS are a consequence of impaired mitochondrial metabolism in the SMA, as shown by the increased levels of angiotensin II type 1 receptor (AGTR1) in the cardiac tissue of these mice (Escobales et al. 2019; Shababi et al. 2010). Myocardial fibrosis has been linked to oxidative stress, but its role in skeletal muscle pathophysiology has been neglected (Shababi et al. 2010).

It was discovered that chemically generated ROS in SMA mice alter *SMN2* splicing, leading to a decrease in the amount of functional SMN protein (Seo et al. 2016). In SMA patients, the combination of low SMN protein levels and oxidative stress resulting from mitochondrial dysfunction reportedly leads to increased cell death, which in turn promotes muscle atrophy and cardiovascular abnormalities (Wijngaarde et al. 2017). In another study, the role of the SMN protein in the regulation of oxidative stress and inflammatory responses in microglia was demonstrated. Specifically, the injection of an antisense oligonucleotide led to a decrease in the expression of oxidative stress markers in the microglia of SMA model mice (Ando et al. 2020). In our study, seven genes involved in oxidative stress mechanisms were investigated, namely, *SOD2, CAT, GPX1, NFE2L2, KEAP1, HMOX1,* and *HMOX2*.

In addition to oxidative stress, inflammation is an important factor in the development and progression of neurodegenerative diseases (Kempuraj et al. 2016). Common features observed in SMA mouse models, such as neurodegeneration, muscle atrophy, cardiovascular pathologies, and diabetes, have been reported to be associated with unresolved systemic inflammation (Simone et al. 2016). For instance, a reanalysis of 154 protein markers derived from the plasma of SMA patients (Kobayashi et al. 2013) revealed that 57 of these markers are associated with inflammatory responses, indicating the presence of systemic inflammation of varying severity in SMA patients (Wan et al. 2018).

The central nervous system is also affected by peripheral inflammation through the activation of microglia by systemic cytokines such as IL-1β and TNF-α (Perry et al. 2007; Qin et al. 2007; Skelly et al. 2013). In human spinal cord samples glial activation and astrogliosis were observed in association with a significant increase in IL-1β and IL-6 (Rindt et al. 2015). These findings, consistent with results from animal models (Deguise et al. 2017; Sahashi et al. 2013; Sintusek et al. 2016; Tarabal et al. 2014; Wan et al. 2018), suggest that neuroinflammation is a common feature of the SMA and likely contributes to the disease phenotype. In addition, a recent study revealed a significant increase in CSF levels of potent proinflammatory cytokines (i.e., IL-6 and TNF-α) in SMA type 1 patients compared to patients with milder forms of the disease (Nuzzo et al. 2023), while another study revealed increased levels of various cytokines (i.e., IL-1β and IL-6) in the blood of pediatric and adult SMA patients (Bonanno et al. 2022). Therefore, nine genes involved in inflammatory processes were included in our study, namely, *GSTP1, IL1B, IL6, IL6R, MIR146A, TNF, NLRP3, CARD8,* and *NOS1*.

Finally, our study included an analysis of genetic variability involving three polymorphisms in the *BDNF* gene and one in the *NOTCH* gene. These polymorphisms, which are associated with neural development, have not been previously studied in the context of SMA. BDNF belongs to a family of proteins crucial for the normal development of both the central and peripheral nervous systems, as well as for neuronal survival and synaptic plasticity processes that play a role in diseases such as SMA (Arancio & Chao 2007). Changes in BDNF levels have been described in the context of SMA (Deng & Chen 2024). NOTCH signaling is a cell-to-cell communication system that is known to regulate the maintenance and differentiation of neural progenitor cells (Louvi & Artavanis-Tsakonas 2006; Yoon & Gaiano 2005). Alterations in NOTCH signaling have been studied in in vitro and in vivo SMA models (Caraballo-Miralles et al. 2013; Maeda et al. 2014; Ohuchi et al. 2019), but the role of genetic variability in the *NOTCH* gene in the pathogenesis of SMA has not yet been described.

In the absence of previously published data, we conducted a case‒control study to investigate whether the genetic variability of selected genes related to oxidative stress, inflammation, or neurodevelopment influences the susceptibility to and course of SMA within a cohort of adult patients with SMA. SMA patients were stratified by SMA type (type 2 and type 3) for comparative analysis, to uncover potential associations between the specific SMA types and the genotype frequencies of the selected genes. We also investigated associations between patient genotype profiles and age of disease symptom onset. Expanding the scope of our study, we examined the effects of genetic variations in selected genes on the motor function, upper limb function, and respiratory function of patients. The aim of this integrative approach was not only to improve the understanding of complex SMA pathology but also to identify patterns that could enable personalized treatment strategies for SMA patients.

## Materials and Methods

In our retrospective cohort study, we enrolled adult Slovenian SMA patients (n = 54) with genetically and clinically confirmed SMA diagnoses for whom comprehensive clinical data were available. Patients were recruited and examined at the Institute of Clinical Neurophysiology, University Medical Centre Ljubljana, Slovenia, between May 2019 and March 2022. Approval of the protocol was granted by the National Medical Ethics Committee of the Republic of Slovenia (NMECRS) (0120–293/2019/8, 120–26/2024–2711-3). Written informed consent was obtained from all participants before inclusion in the study.

Demographic and clinical data, including sex, SMA type, *SMN1* gene mutation, *SMN2* copy number, age at blood sampling, and age at symptom onset, were included in the analyses concerning the associations between the studied polymorphisms and susceptibility to SMA, SMA type, and age at symptom onset. In addition, motor, upper limb, and respiratory functions were assessed during routine examinations. For the analyses, data from examinations performed at time points proximal to blood collection were used to ensure consistency of clinical measurements with the time frame of blood collection. The motor functions of the SMA patients were assessed based on the Revised Hammersmith Scale (RHS) and the Revised Upper Limb Module (RULM) by qualified clinical assessors following the center’s clinical practice. The outcomes assessed during the study included pulmonary function test results, i.e., vital capacity (VC) and peak expiratory flow (PEF). Both the raw values and the predicted percentages were given for spirometry. Disease duration, calculated as the interval between the reported age at symptom onset and the age at blood sampling, was recorded.

A control group consisting of 163 unrelated healthy blood donors without neurological diseases was included in the study to assess susceptibility to SMA. Information on sex and age at the time of blood sampling was available for the control group. The protocol received approval from the NMECRS (60/02/02, 43/02/09).

The genotyping of 29 specific single nucleotide polymorphisms (SNPs) in 18 genes associated with oxidative stress, inflammatory processes, and neurodevelopmental mechanisms was conducted using competitive allele-specific polymerase chain reaction (KASP). Of the 18 genes analyzed, 7 are involved in oxidative stress pathways (i.e., *SOD2, CAT, GPX1, NFE2L2, KEAP1, HMOX1, and HMOX2*), 9 are involved in inflammatory pathways (i.e., *GSTP1, IL1B, IL6, IL6R, MIR146A, TNF, NLRP3, CARD8, and NOS1*), and 2 are involved in neurodevelopment (i.e., *BDNF and NOTCH*). All selected polymorphisms were considered functional genetic variants with a minor allele frequency of 5% or more. The characteristics of the genetic variants analyzed in this study are listed in Table S1. In addition, homozygous deletions of the *GSTM1* and *GSTT1* genes were detected. The selection of specific polymorphisms was based on the analysis of previous research findings, which indicated the potential significance of these genetic variants in the pathogenesis of similar neurodegenerative diseases (Atanasovska Velkovska et al. 2021; Ravnik-Glavač et al. 2022; Vogrinc et al. 2023).

The isolation of genomic DNA from peripheral blood samples was performed following the manufacturer's recommended protocol using the MagMAX™-96 DNA Multi-Sample Kit (Thermo Fisher Scientific, Waltham, MA, USA) on the KingFisher Duo Prime sample preparation instrument (Thermo Fisher Scientific, Waltham, MA, USA).

DNA quantity and purity were determined spectrophotometrically using Lambda Bio (PerkinElmer, Waltham, MA, USA). DNA concentration was measured at 260 nm, and the ratio of absorbance values at 260 nm and 280 nm was used to estimate DNA purity (Table S9). The quality of the DNA was determined by gel electrophoresis, with 10% of the samples analyzed on a 1% agarose gel to verify the integrity of the DNA.

Following the manufacturer's instructions (KBiosciences, Herts, UK, and LGC Genomics, UK), the DNA was diluted to 15 ng/µl. For KASP genotyping, 6 µl of KASP master mix, which included 0.11 µl of the custom KASP genotyping assay (KASP Assays, LGC Biosearch Technologies, Hoddesdon, UK), was added to each well containing 2 µl of DNA.

Deletion analysis of the *GSTT1* and *GSTM1* genes was conducted using a multiplex PCR method that detects homozygous deletions, as previously described (Chen et al. 1996). In brief, the *GSTT1* and *GSTM1* genes were amplified simultaneously via a one-step PCR together with the β-globin (*HBB*) gene, which served as an internal positive control. The primer pairs used were as follows: 5'-CTGCCCTACTTGATTGATGGG-3' and 5'-CTGGATTGTAGCAGATCATGC-3' for *GSTM1*; 5'-TTCCTTACTGGTCCTCACATCTC-3' and 5'-TCACCGGATCATGGCCAGCA-3' for *GSTT1*; 5'-ACACAACTGTGTTCACTAGC-3'; and 5'-CAACTTCATCCACGTTCACC-3' for *HBB* (Sangon Biotech, Shanghai, China). Parts of the PCR products were analyzed on a 2% agarose gel to identify the *GSTM1*-specific 600 bp fragment, the *GSTT1*-specific 480 bp fragment, and the *HBB*-specific 268 bp fragment. To ensure the reliability of the genotyping results, genotyping was performed in duplicate on 10% of the randomly selected samples.

Population characteristics are expressed as medians and interquartile ranges (25–75%) for continuous variables or as frequencies for categorical variables. If there were more than two samples in the group, the interquartile range was calculated using the weighted averages; if there were only two samples in the group, Tukey's hinge was used. The genotype frequencies of the control group were compared with those of Hardy‒Weinberg equilibrium (HWE) using the chi-square test. The Shapiro‒Wilk test was used to assess the conformity of the numerical variables to a normal distribution. For the comparison of continuous and categorical variables between SMA patients and controls, the nonparametric Mann‒Whitney test and Fisher's exact test, respectively, were used.

Logistic regression was used to examine the associations between specific polymorphisms and binary categorical variables, and the odds ratios (ORs) and corresponding 95% confidence intervals (CIs) were calculated. When one of the groups had no subjects, Fisher's exact test was used instead. The analysis included three genetic models: additive, dominant, and recessive models. The nonparametric Mann‒Whitney test and Kruskal‒Wallis test were used to assess the association of polymorphisms with RHS, RULM, VC (%), and PEF (%). ANCOVA was used to adjust for clinical variables, namely, age at blood collection, *SMN2* copy number, and disease duration.

Each statistical test was two-sided. Bonferroni corrections were applied for pairwise comparisons to mitigate the risk of false positive results. The threshold for statistical significance was set at 0.05, except for the genetic data, for which the threshold for significance was set at 0.0016 (0.05/31), with *p* values less than 0.0016 considered to indicate statistical significance and *p* values between 0.0016 and 0.050 considered nominally significant. All analyses were conducted with the statistical software IBM SPSS Statistics, version 27.0 (IBM Corporation, Armonk, NY, USA).

To compare the genotype frequencies of allele variants with an average minor allele frequency (MAF) of 0.29 between 54 patients and 163 controls, our study had a power of 80% to detect odds ratios (ORs) of 0.305 or less and ORs of 2.455 or more. Power calculations were performed using PS Power and sample size calculations, version 3.1.6 (Dupont & Plummer 1990).

## Results

We enrolled 54 SMA patients and 163 healthy controls in our study. The demographic and clinical characteristics of the patients are presented in Table 1. The data showed that 10 (18.5%) SMA patients had a homozygous deletion of exon 7 in *SMN1*, 43 (79.6%) had a homozygous deletion of exon 7 and exon 8 in *SMN1*, and 1 (1.9%) had a homozygous deletion of exon 7 and exon 8 and a point mutation in *SMN1*. Among the patients, the majority had three (26, 53.1%) or four (20, 40.8%) copies of the *SMN2* gene. One patient (2.0%) had two copies, and two patients (4.1%) had five copies of the *SMN2* gene. Additionally, patients were classified as SMA type 2 (24, 46.2%), type 3 (25, 48.1%), or type 4 (3, 5.8%). Data on SMA type were missing for 2 patients, and data regarding the copy number of the *SMN2* gene were not available for 5 patients. The median age at disease onset was 3.9 (1.3–11.0) years. At the beginning of the study, all patients were treatment naïve. One patient died at the time of analysis.

**Table 1:** Overview of demographic and clinical parameters of SMA patients

Patient’s Characteristic	Category/Unit	SMA Cohort (N = 54)	SMA Type 2 (N = 24)	SMA Type 3 (N = 25)
Sex	Male, N (%)	26 (48.1)	9 (37.5)	15 (60.0)
	Female, N (%)	28 (51.9)	15 (62.5)	10 (40.0)
SMA Type	2, N (%)	24 (46.2) [2]	/	/
	3, N (%)	25 (48.1)	/	/
	4, N (%)	3 (5.8)	/	/
Number of *SMN2* copies	2, N (%)	1 (2.0) [5]	0 [3]	0 [2]
	3, N (%)	26 (53.1)	17 (81.0)	8 (34.8)
	4, N (%)	20 (40.8)	4 (19.0)	14 (60.9)
	5, N (%)	2 (4.1)	0	1 (4.3)
Age at the time of blood collection for genotyping	Median (25–75%), years	42.5 (27.0–52.3)	30 (23.50–44.50)	51.0 (41.5–54.0)
Age at symptom onset	Median (25–75%), years	3.0 (1.3–11.0) [3]	1.30 (0.70–1.50) [1]	10.0 (3.25–14.75) [1]
Disease duration	Median (25–75%), years	33.0 (24.0–45.0) [3]	28.0 (22.0–37.0) [1]	239.5 (32.3–48.3) [1]
RHS	Median (25–75%)	4.00 (1.25–16.5) [2]	2.00 (0–4.00) [1]	8.00 (2.00–32.5)
RULM	Median (25–75%)	18.0 (9.00–25.0) [3]	15.0 (9.00–22.0) [1]	20.5 (9.50–31.3) [1]
VC (%)	Median (25–75%), %	59.0 (38.0–87.8) [2]	38.0 (23.0–59.0) [1]	81.0 (58.0–92.0)
PEF (%)	Median (25–75%), %	60.0 (47.0–85.5) [2]	50 (36.0–64.0) [1]	77.0 (51.5–87.5)

SMA type 4 patients (n = 3) are not represented in a separate column. The number of missing data points is presented in brackets. *SMA* spinal muscular atrophy; *SMN2*: survival of motor neuron 2; *RHS* Revised Hammersmith Scale; *RULM* Revised Upper Limb Module; *VC* vital capacity; *PEF* peak expiratory flow

The control group consisted of 113 (69.3%) males and 50 (30.7%) females; i.e., there were significantly more male participants in the control group than in the SMA cohort (males: 48.1%, females: 51.9%, *p* = 0.008). The median ages of the SMA patients and healthy controls at the time of blood sampling were 42.5 (27–52.3) years and 45 (40–49) years, respectively. The age distribution did not differ significantly between the groups (*p* = 0.260).

The genotype frequencies of the selected polymorphisms, MAFs, and data confirming the concordance of the genotype distribution of the control cohort with that of HWE are shown in Table S1.

Three of the 31 genetic variants analyzed were significantly associated with susceptibility to SMA (Table 2). Although univariate logistic regression analysis did not yield statistically significant results, nominally significant results indicated a possible association between SMA risk and the SNPs *IL6* rs1800795, *TNF* rs1800629, and *BDNF* rs6265. The entire dataset with all analyzed polymorphisms is presented in Table S2.

**Table 2:** Comparisons of nominally significant genotype frequencies between SMA patients and controls

Gene	SNP	Genotype	Controls, N (%)	SMA, N (%)	OR (95% CI)	*p*-value
*IL6*	*rs1800795	GG	65 (39.9)	10 (18.5)	Reference	
		GC	67 (41.1)	29 (39.2)	2.813 (1.270–6.234)	**0.011**
		CC	31 (19.0)	15 (27.8)	3.145 (1.269–7.793)	**0.013**
		GC + CC	98 (60.1)	44 (81.5)	2.918 (1.372–6.208)	**0.005**
*TNF*	rs1800629	GG	106 (65)	43 (79.6)	Reference	
		GA	53 (32.5)	9 (16.7)	0.419 (0.190–0.923)	**0.031**
		AA	4 (2.5)	2 (3.7)	1.233 (0.218–6.980)	0.813
		GA + AA	57 (35)	11 (20.4)	0.476 (0.228–0.993)	**0.048**
*BDNF*	rs6265	CC	103 (63.2)	41 (75.9)	Reference	
		CT	53 (32.5)	9 (16.7)	0.427 (0.193–0.944)	**0.035**
		TT	7 (4.3)	4 (7.4)	1.436 (0.399–5.167)	0.580
		CT + TT	60 (36.8)	13 (24.1)	0.544 (0.270–1.097)	0.089

^*^A recessive model was used. *SMA* Spinal muscular atrophy; *SNP* single nucleotide polymorphism; *OR* odds ratio; *CI* confidence interval. Nominally significant results are shown in bold

GC heterozygotes (GG/GC, OR = 2.813, 95% CI = 1.270–6.234, *p* = 0.011) and CC homozygotes (GG/CC, OR = 3.145, 95% CI = 1.269–7.793, *p* = 0.013) for the *IL6* polymorphism rs1800795 had almost threefold and more than threefold greater odds of SMA, respectively. The association remained nominally significant in carriers of at least one *IL6* rs1800795 C allele (GG/GC + CC, OR = 2.918, 95% CI = 1.372–6.208, *p* = 0.005). In contrast, there were significant associations between the GA heterozygotes for *TNF* rs1800629 (GG/GA, OR = 0.419, 95% CI = 0.190–0.923, *p* = 0.031) and carriers of at least one A allele (GG/GA + AA, OR = 0.476, 95% CI = 0.228–0.993, *p* = 0.048) and lower SMA risk according to the additive and dominant genetic models, respectively. Similarly, carriers of the *BDNF* rs6265 CT genotype (CC/CT, OR = 0.427, 95% CI = 0.193–0.944, *p* = 0.035) had decreased odds of having SMA.

The results of both univariate and multivariate analyses examining the associations between selected polymorphisms and SMA type are presented in Table 3. Four variants, namely, *NFE2L2* rs35652124, *HMOX2* rs1051308, *CARD8* rs2043211, and *BDNF* rs6265, had nominally significantly different genotype frequencies between the patients with SMA type 2 and those with SMA type 3.

**Table 3:** Comparison of nominally significant genotype frequencies among patients with SMA types 2 and 3 (n = 44)

Gene	SNP	Genotype	SMA Type 2 (N = 21)	SMA Type 3 (N = 23)	OR (95% CI)	*p*-value	OR_adj_ (95% CI)	*p*_adj_-value
*NFE2L2*	rs35652124	TT	13 (61.9)	7 (30.4)	Reference			
		TC	6 (28.6)	12 (52.2)	3.714 (0.969–14.23)	0.056	2.569 (0.589–11.19)	0.209
		CC	2 (9.5)	4 (17.4)	3.714 (0.539–25.59)	0.183	1.654 (0.177–15.50)	0.659
		TC + CC	8 (38.1)	16 (69.6)	3.714 (1.063–12.98)	**0.040**	2.336 (0.586–9.319)	0.229
*HMOX2*	*rs1051308	AA	15 (71.4)	8 (34.8)	Reference			
		AG	6 (28.6)	14 (60.9)	4.375 (1.210–15.81)	**0.024**	3.411 (0.836–13.92)	0.087
		GG	0	1 (4.3)	/	0.033**	/	/
		AG + GG	6 (28.6)	15 (65.2)	4.687 (1.306–16.82)	**0.018**	3.552 (0.873–14.45)	0.077
*CARD8*	rs2043211	AA	15 (71.4)	6 (26.1)	Reference			
		AT	4 (19.0)	15 (65.2)	9.375 (2.191–40.11)	**0.003**	7.639 (1.563–37.35)	**0.012**
		TT	2 (9.5)	2 (8.7)	2.500 (0.284–22.04)	0.409	1.901 (0.169–21.34)	0.603
		AT + TT	6 (28.6)	17 (73.9)	7.083 (1.878–26.72)	**0.004**	5.669 (1.328–24.19)	**0.019**
*BDNF*	rs6265	CC	15 (71.4)	21 (91.3)	Reference			
		CT	4 (19.0)	2 (8.7)	0.357 (0.058–2.209)	0.268	0.184 (0.019–1.794)	0.145
		TT	2 (9.5)	0	/	0.126**	/	/
		CT + TT	6 (28.6)	2 (8.7)	0.238 (0.042–1.346)	0.104	0.112 (0.013–0.974)	**0.047**

^*^A recessive model was used. **Calculated using Fisher’s exact test. *Adj* adjusted for *SMN2* copy number; *SMA* Spinal muscular atrophy; *SNP* single nucleotide polymorphism. Nominally significant results are printed in bold text

Individuals who were heterozygous for *CARD8* rs2043211 (AA/AT, OR = 9.375, 95% CI = 2.191–40.11, *p* = 0.003) or who were carriers of at least one *CARD8* rs2043211 T allele (AA/AT + TT, OR = 7.083, 95% CI = 1.878–26.72, *p* = 0.004) had increased odds of having SMA type 3. After adjusting for the number of *SMN2* copies, the associations remained nominally significant (AA/AT, OR = 7.639, 95% CI = 1.563–37.35, *p* = 0.012; AA/AT + TT, OR = 5.669, 95% CI = 1.328–24.19, *p* = 0.019). Similarly, heterozygotes for the *HMOX2* polymorphism rs1051308 (AA/AG, OR = 4.375, 95% CI = 1.210–15.81, *p* = 0.024) and GG homozygotes (*p* = 0.033**) had increased odds of having SMA type 3. Furthermore, individuals carrying at least one *HMOX2* rs1051308 G allele (AA/AG + GG, OR = 4.687, 95% CI = 1.306–16.82, *p* = 0.018) or at least one *NFE2L2* rs35652124 C allele (TT/TC + CC, OR = 3.714, 95% CI = 1.063–12.98, *p* = 0.040) had higher odds of having SMA type 3 than type 2. After adjusting for the number of *SMN2* copies, the associations between the *HMOX2* rs1051308 and *NFE2L2* rs35652124 polymorphisms and the SMA type were no longer significant.

Conversely, carriers of at least one polymorphic *BDNF* rs6265 T allele had lower odds of having SMA type 3 when adjusting for the number of *SMN2* copies (CC/CT + TT, OR = 0.112, 95% CI = 0.093–0.574, *p* = 0.047). The associations between SMA type and all the genetic variants studied are shown in Table S3.

We hypothesized that the variability of selected genes influences the age at which a patient develops the first symptoms of the disease. The analysis revealed nominally significant associations between the polymorphisms *NFE2L2* rs35652124, *TNF* rs1800629, *HMOX2* rs1051308, and rs2270363 and age at symptom onset (Table 4).

**Table 4:** Associations between nominally significant genetic variants and age of symptom onset (n = 46)

Gene	SNP	Genotype	SMA N (%)	Age at symptom onset, Median (25–75%)	*p*-value	*p*_adj_-value
*SOD2*	rs4880	AA	11 (23.9)	3.00 (0.90–16.00)	0.964	0.067
		AG	23 (50.0)	3.00 (1.00–12.00)		
		GG	12 (26.1)	3.00 (1.08–7.00)		
		AG + GG	35 (76.1)	3.00 (1.00–10.00)	0.800	**0.021**
*CAT*	rs1001179	CC	22 (47.8)	3.00 (0.94–13.00)	0.944	**0.014**
		CT	20 (43.5)	3.00 (1.26–10.00)		
		TT	4 (8.7)	5.30 (0.53–31.00)		
		CT + TT	24 (52.2)	3.00 (1.06–10.00)	0.851	0.568
*NFE2L2*	rs35652124	TT	20 (43.5)	1.75 (1.00–3.75)	0.128	0.420
		TC	20 (43.5)	6.00 (0.99–15.50)		
		CC	6 (13.0)	8.00 (1.30–15.25)		
		TC + CC	26 (56.5)	7.00 (1.29–15.25)	**0.047**	0.563
*HMOX2*	*rs1051308	AA	24 (52.2)	1.40 (1.00–5.50)	**0.023**	0.285
		AG	20 (43.5)	4.00 (2.00–14.50)		
		GG	2 (4.3)	23.00 (20.00–26.00)		
		AG + GG	22 (47.8)	5.00 (2.00–16.00)	**0.031**	0.225
	*rs2270363	GG	29 (63.0)	2.00 (1.00–6.00)	**0.048**	0.475
		GA	15 (32.6)	4.00 (1.95–14.50)		
		AA	2 (4.3)	23.00 (20.00–26.00)		
		GA + AA	17 (37.0)	10.00 (1.95–16.00)	0.074	0.622
*MIR146A*	*rs2910164	GG	25 (54.3)	2.00 (1.00–7.00)	0.096	0.055
		GC	18 (39.1)	4.00 (1.30–16.00)		
		CC	3 (6.5)	8.00 (6.00–24.00)		
		GC + CC	21 (45.7)	4.00 (1.30–18.00)	0.059	**0.020**
*TNF*	rs1800629	GG	36 (78.3)	3.00 (1.13–7.50)	**0.031**	0.530
		GA	8 (17.4)	11.00 (5.75–20.00)		
		AA	2 (4.3)	0.70 (0.50–0.90)		
		GA + AA	10 (21.7)	10.00 (0.85–20.00)	0.350	0.441

^*^A recessive model was used. *Adj* adjusted for *SMN2* copy number; *SMA* Spinal muscular atrophy; *SNP* single nucleotide polymorphism. Nominally significant results are printed in bold text

In AA homozygotes for *HMOX2* rs1051308 (AA/GG, *p* = 0.023) or GG homozygotes for *HMOX2* rs2270363 (GG/AA, *p* = 0.048), the onset of disease symptoms occurred at a nominally significantly younger age. Moreover, the association between *HMOX2* rs1051308 and earlier symptom onset was nominally significant according to the recessive model (AA/AG + GG, *p* = 0.031). In contrast, heterozygotes for *TNF* rs1800629 (GG/GA, *p* = 0.031) or carriers of at least one *NFE2L2* rs35652124 C allele (TT/TC + CC, *p* = 0.047) were found to be nominally significantly associated with a later onset of symptoms. However, after adjusting for *SMN2* copy number, these associations did not retain their significance (Table 4).

After accounting for the *SMN2* copy number, the SNPs *SOD2* rs4880, *CAT* rs1001179, and *MIR146A* rs2910164 showed a nominally significant effect on the age of onset of symptoms. Individuals carrying at least one *SOD2* rs4880 G allele (AA/AG + GG, *p* = 0.021) or at least one *MIR146A* rs2910164 C allele (GG/GC + CC, *p* = 0.020) developed symptoms nominally significantly later than AA homozygotes or GG homozygotes, respectively. No significant or nominally significant associations were found in the additive models for either *SOD2* rs4880 or *MIR146A* rs2910164. In TT homozygotes for *CAT* rs1001179, a nominally significantly later symptom onset was observed (CC/TT, *p* = 0.014), but no significant associations were found in the additive model. The results for each genetic variant analyzed are shown in Table S4.

One aim of this study was to investigate the effects of selected genetic variants on patients' motor function and upper limb function, as measured by RHS and RULM scores, respectively. The results of the analyses revealed a nominally significant associations between the RHS and the five polymorphisms (Table 5).

**Table 5:** Associations between nominally significant polymorphisms and the RHS (n = 45)

Gene	SNP	Genotype	RHS score, Median (25–75%)	*p*-value	*p*_adj_-value
*GPX1*	rs1050450	GG	3.00 (1.00–8.00)	0.068	0.132
		GA	7.00 (4.00–37.50)		
		AA	0 (0–21.00)		
		GA + AA	6.50 (2.75–42.50)	0.083	**0.044**
*NFE2L2*	*rs6721961	GG	8.00 (2.75–42.50)	**0.025**	0.172
		GT	2.00 (0–4.25)		
		GT + TT	2.00 (0–4.00)	**0.007**	0.064
*HMOX1*	rs2071747	GG	5.00 (2.00–29.00)	0.286	**0.006**
		GC + CC	3.00 (1.50–5.25)		
*MIR146A*	*rs2910164	GG	4.00 (0.50–8.50)	0.150	0.107
		GC	4.00 (2.00–48.00)		
		CC	29.00 (17.50–37.00)		
		GC + CC	6.00 (2.00–46.50)	0.117	**0.034**
*NOTCH*	rs367398	GG	2.00 (0–7.50)	**0.028**	**0.037**
		GA	14.00 (3.00–44.00)		
		AA	4.00 (1.50–5.75)		
		GA + AA	5.00 (2.50–36.50)	**0.025**	**0.050**

^*^A recessive model was used. *Adj* adjusted for age, *SMN2* copy number and disease duration. *RHS* Revised Hammersmith scale; *SMA* Spinal muscular atrophy; *SNP* single nucleotide polymorphism. Nominally significant results are printed in bold text

Regarding *NFE2L2* rs6721961, heterozygotes (GG/GT, *p* = 0.025) and carriers of at least one T allele (GG/GT + TT, *p* = 0.007) exhibited lower RHS scores. Conversely, both *NOTCH* rs367398 heterozygotes (GG/GA, *p* = 0.028) and carriers of at least one A allele (GG/GA + AA, *p* = 0.025) demonstrated higher RHS scores. Remarkably, the results for *NOTCH* rs367398 remained nominally significant even after we adjusted for age, *SMN2* copy number, and disease duration in both the additive (GG/GA, *p* = 0.037) and dominant (GG/GA + AA, *p* = 0.050) models. After accounting for the above clinical parameters, individuals carrying at least one *GPX1* rs1050450 A allele (GG/GA + AA, *p* = 0.044) or at least one *MIR146A* rs2910164 C allele (GG/GC + CC, *p* = 0.034) were nominally significantly associated with higher RHS scores. No significant or nominally significant associations were detected in the additive models. On the other hand, ANCOVA showed that carriers of at least one *HMOX1* rs2071747 C allele (GG/GC + CC, *p* = 0.006) exhibited nominally significantly lower RHS scores. Table S5 shows the results for all the genetic variants examined.

In terms of upper limb function, the analysis revealed the nominal significance of the association between the RULM score and three polymorphisms, as shown in Table 6. Increased RULM scores were observed in carriers of at least one polymorphic *MIR146A* rs2910164 C allele (GG/GC + CC, *p* = 0.036), and the results remained nominally significant after adjustment for age, *SMN2* copy number, and disease duration (*p* = 0.018). The additive models showed no significant or nominally significant associations. Similarly, heterozygotes (GG/GA, *p* = 0.047) and carriers of at least one *NOTCH rs367398* A allele (GG/GA + AA, *p* = 0.022) had higher RULM scores. The association remained nominally significant even after adjustment (GG/GA,* p* = 0.031 and GG/GA + AA, *p* = 0.011). In contrast, *GSTM1* deletion was associated with nominally significantly lower RULM scores after adjustment for clinical parameters (*p* = 0.030). Table S6 shows the results for each genetic variation analyzed.

**Table 6:** Associations between nominally significant polymorphisms and RULM score (n = 44)

Gene	Polymorphism	Genotype	RULM score, Median (25–75%)	*p*-value	*p*_adj_-value
*MIR146A*	*rs2910164	GG	18.00 (9.00–23.00)	0.070	0.062
		GC	21.50 (14.00–36.00)		
		CC	24.00 (23.50–30.50)		
		GC + CC	23.00 (14.50–36.50)	**0.036**	**0.018**
*NOTCH*	rs367398	GG	14.50 (9.00–18.75)	**0.047**	**0.031**
		GA	23.00 (14.75–34.50)		
		AA	22.50 (5.25–25.50)		
		GA + AA	23.00 (14.25–32.75)	**0.022**	**0.011**
*GSTM1*-present			23.50 (13.75–33.25)	0.107	**0.030**
*GSTM1*-null			18.00 (8.75–24.00)		

^*^A recessive model was used. *Adj* adjusted for age, *SMN2* copy number and disease duration. *RULM* Revised Upper Limb Module. Nominally significant results are printed in bold text

We investigated the effects of selected polymorphisms on the pulmonary function status of SMA patients. Among the investigated polymorphisms, associations between changes in pulmonary function and genotype frequencies were found for several SNPs, and the results are shown in Table 7 and Table 8.

**Table 7:** Associations between nominally significant polymorphisms and VC (%) (n = 45)

Gene	SNP	Genotype	VC (%), Median (25–75%)	*p*-value	*p*_adj_-value
*HMOX1*	rs2071747	GG	67.00 (38.00–91.00)	0.568	**0.028**
		GC + CC	56.50 (36.25–85.50)		
*BDNF*	rs6265	CC	67.00 (40.00–87.00)	0.800	0.099
		CT	42.00 (23.00–96.00)		
		TT	72.50 (10.00–135.00)		
		CT + TT	42.00 (13.25–106.50)	0.590	**0.032**
*NOTCH*	rs367398	GG	52.50 (24.00–73.25)	**0.049**	0.135
		GA	85.00 (40.00–98.00)		
		AA	53.00 (33.50–74.00)		
		GA + AA	80.00 (41.00–94.50)	0.064	0.084

*Adj* adjusted for age, SMN2 copy number and disease duration. SNP: single nucleotide polymorphism; VC (%): vital capacity percent predicted. Nominally significant results are printed in bold text

**Table 8:** Associations between nominally significant polymorphisms and PEF (%) (n = 45)

Gene	SNP	Genotype	PEF (%), Median (25–75%)	*p*-value	*p*_adj_-value
*GPX1*	rs1050450	GG	52.00 (46.50–76.00)	0.183	0.112
		GA	80.00 (52.50–85.00)		
		AA	42.00 (30.50–81.00)		
		GA + AA	78.50 (50.75–87.75)	0.122	**0.045**
*NFE2L2*	*rs6721961	GG	78.50 (50.00–90.00)	0.132	0.598
		GT	51.50 (45.00–65.00)		
		GT + TT	51.00 (46.00–64.00)	**0.047**	0.319
	rs35652124	TT	51.50 (40.50–68.50)	**0.036**	0.123
		TC	68.00 (47.00–86.00)		
		CC	86.50 (77.50–101.75)		
		TC + CC	80.00 (49.00–87.50)	0.093	0.747
*HMOX1*	rs2071747	GG	64.00 (50.00–86.00)	0.257	**0.009**
		GC + CC	49.00 (38.25–73.25)		
*NOTCH*	rs367398	GG	49.00 (40.00–80.00)	0.067	0.212
		GA	77.00 (53.00–93.00)		
		AA	56.50 (39.75–77.50)		
		GA + AA	68.00 (51.00–89.50)	**0.044**	0.094

^*^A recessive model was used. *Adj* adjusted for age, *SMN2* copy number and disease duration. PEF (%): peak expiratory flow percent predicted; *SNP* single nucleotide polymorphism. Nominally significant results are printed in bold text

Nominally significantly greater VC (%) values were observed in heterozygotes for *NOTCH* rs367398 (GG/GA, *p* = 0.049), but no significant effect on VC (%) was observed after adjustment for age, *SMN2* copy number, or disease duration. After adjustment for the above clinical parameters, carriers of at least one *HMOX1* rs2071747 C allele (GG/GC + CC, *p* = 0.028) or carriers of at least one *BDNF* rs6265 T allele (CC/CT + TT, *p* = 0.032) had lower VC (%) values.

In terms of PEF (%) values, carriers of at least one *NOTCH* rs367398 A allele (GG/GA + AA, *p* = 0.044) had higher PEFs (%), and carriers of at least one *NFE2L2* rs6721961 T allele (GG/GT + TT, *p* = 0.047) had lower PEFs (%). Increased PEF (%) values were observed in CC homozygotes for *NFE2L2* rs35652124 (TT/CC, *p* = 0.036); however, the association was not significant in the dominant model. After adjustment for clinical parameters, none of the associations remained significant. On the other hand, nominally significant results were observed for the polymorphisms *GPX1* rs1050450 and *HMOX1* rs2071747 after accounting for age, *SMN2* copy number, and disease duration. Carriers of at least one *GPX1* rs105045 A allele (GG/GA + AA, *p* = 0.045) were associated with higher PEF (%), while carriers of at least one *HMOX1* rs2071747 C allele (GG/GC + CC, *p* = 0.009) were associated with lower PEF (%). The complete data on the influence of the selected polymorphisms on lung function status are shown in Table S7 and Table S8.

## Discussion and Conclusions

The present study explored the relationships between selected functional genetic variants in genes associated with oxidative stress, inflammation, and neurodevelopment and susceptibility to SMA, SMA type, and age at symptom onset. For the subgroup analysis, patients were classified into SMA type 2 and type 3 groups, which allowed more robust analyses aimed at revealing the genetic factors influencing the severity of SMA. However, the SMA type 4 group, which included only three subjects, significantly limited the significance of the analyses due to the small sample size, resulting in the exclusion of these subjects. To account for the influence of the *SMN2* copy number, which is known to correlate with SMA type and age at disease onset, the results were adjusted accordingly. In addition, we investigated the associations of these genetic variations with RHS scores, assessing patients' motor function, and with RULM scores, focusing specifically on the assessment of upper limb function. Finally, we aimed to evaluate the impact of the selected polymorphisms on lung function status.

To date, no studies have investigated the impact of the genetic variability of the genes examined in our study, particularly concerning the role of inflammation, the oxidative stress response, and neurodevelopment in the etiopathogenesis of the SMA. Thus, this study provides important new insights into this topic. Our results showed that *TNF* rs1800629 and *BDNF* rs6265 had protective effects on susceptibility to SMA. In contrast, the *IL6* polymorphism rs1800795 was associated with an increased risk of SMA. The observed associations between polymorphisms in the *NFE2L2, HMOX2, CARD8,* and *BDNF* genes and SMA type and between polymorphisms in the *SOD2, CAT, NFEL2L2, HMOX2, MIR146A,* and *TNF* genes and age at onset of symptoms underscore the importance of the above pathways in SMA. In addition, the associations of polymorphisms in the *GPX1, NFE2L2, HMOX1, MIR146A, NOTCH, GSTM1,* and *BDNF* genes with RHS, RULM, VC (%), or PEF (%) values highlight the potential relevance of specific pathways to the disease course. In interpreting our results, it should be noted that these associations were only nominally significant, suggesting that further investigation and validation are required to ascertain the role of oxidative stress, inflammation, and neurodevelopment in the SMA.

Among the polymorphisms in the inflammatory pathways, we observed a protective effect against susceptibility to SMA in heterozygotes and carriers of at least one A allele for the *TNF* rs1800629 polymorphism. This was also consistent with the observation that heterozygosity for the *TNF* rs1800629 A allele was associated with a later onset of symptoms. The *TNF* rs1800629 polymorphism, located in the 5' promoter region of the *TNF* gene, results in two types of alleles. The first is a common allele defined by the presence of G, and the second is a rarer allele defined by the substitution of A for G at position -308 (Wilson et al. 1992). The *TNF* rs1800629 polymorphism is known to affect the expression of TNF-α (Abdalhabib et al. 2022; Elahi et al. 2009; Ibrahim et al. 2012; Šedý et al. 2014; Takeuchi et al. 2002). In addition, it plays a role in neurodegeneration and may also affect hippocampal volume in individuals without known neurological diseases (Baune et al. 2012). To date, no studies have been conducted on the *TNF* rs1800629 polymorphism in the context of SMA. However, studies have reported increased production of TNF-α in SMN-depleted RAW264.7 (Ando et al. 2020) and SMN-deficient BV2 cells (Kim & Choi 2017). On the contrary, Kobayashi et al. (2023) analyzed TNF-α levels in CSF to assess their potential association with symptoms and treatment response in pediatric SMA patients and found that TNF-α levels remained unchanged (Kobayashi et al. 2023). Consistent with our results, the protective effect of the rs1800629 minor A allele has been described in several diseases (Areeshi et al. 2017; Hamadien et al. 2016), including Alzheimer’s disease (AD) (Sarajärvi et al. 2010; Vogrinc et al. 2023).

We also found a protective effect against susceptibility to SMA in heterozygotes for *BDNF* rs6265. However, individuals carrying at least one polymorphic *BDNF* rs6265 T allele exhibited lower odds of having SMA type 3 than SMA type 2 when the number of *SMN2* copies was taken into account. The *BDNF* rs6265 variant, located in the prodomain region of the *BDNF* gene, leads to the substitution of valine (Val) with methionine (Met) at codon 66 (p.Val66Met) of the proBDNF protein as a result of a nucleotide change from C to T at position 196. Although the rs6265 polymorphism is classified as a benign polymorphism according to the American College of Medical Genetics and Genomics (ACMG) guidelines (Richards et al. 2015), it is considered a potential risk factor because the presence of Met may be associated with decreased activity-dependent BDNF secretion (Egan et al. 2003). At the mechanistic level, *BDNF* rs6265 is thought to affect not only BDNF production but also BDNF localization and signal transduction, resulting in subtle phenotypic changes that affect MNs and neuromuscular junctions (Nguyen et al. 2023).

To date, several studies have shown conflicting results on the associations between BDNF levels in control subjects and individuals with the *BDNF* rs6265 polymorphism (Lang et al. 2009; Ozan et al. 2010; Yoshimura et al. 2011). Although studies on various diseases, including neurological diseases (Bian et al. 2005), have shown an increased risk of disease associated with the rs6265 polymorphism, there is some evidence suggesting a protective role of this *BDNF* variant against cognitive impairment in individuals with multiple sclerosis (MS) (Portaccio et al. 2021). Another study in a large cohort of MS patients showed that the rs6265 SNP is associated with improved brain structure and function (Zivadinov et al. 2007). Similarly, studies in AD patients have shown that homozygosity for the Val allele confers an increased risk of AD, suggesting that the Met allele has a protective effect (Matsushita et al. 2005; Ventriglia et al. 2002).

Our results also demonstrated an association between the *IL6* rs1800795 C allele and an increased risk of SMA. The *IL6* rs1800795 polymorphism is a C-to-G change located in the promoter region of the *IL-6* gene at position -174 and frequently exhibits polymorphic traits in Caucasians. Individuals with the *IL6* rs1800795 CC genotype were found to have elevated levels of IL-6 in both blood and brain tissue and were associated with AD. In addition, this genotype was overrepresented in AD patients compared to controls, which significantly increased the risk of developing AD (Licastro et al. 2003). In a study on the risk of MS in a Polish population, researchers found significantly more carriers of the C allele in the MS group than in healthy individuals (Mirowska-Guzel et al. 2011). Finally, a study by Fontalba et al. (2009) showed that the *IL6* rs1800795 and *CARD8* rs2043211 polymorphisms exhibit synergistic effects leading to a sixfold reduction in AD risk in individuals who simultaneously possess the *IL6* rs1800795 CC genotype and the *CARD8* rs2043211 TT genotype (Fontalba et al. 2009).

Consistent with the notion of a protective role of the *CARD8* rs2043211 T allele, our results showed that heterozygotes or carriers of at least one polymorphic *CARD8* rs2043211 T allele were more likely to be associated with milder type 3 SMA. Proteins containing the caspase recruitment domain (CARD) motif are generally associated with the activation of NF-κB, an important transcription factor for the control of inflammation. However, in response to proinflammatory signals, CARD8, unlike most other CARD proteins documented to date, significantly inhibits NF-κB activation (Bouchier-Hayes et al. 2001; Razmara et al. 2002). The *CARD8* rs2043211 polymorphism involves a change from T to A at the third nucleotide of codon 10. This change leads to the formation of a premature stop codon (allele A), resulting in significant truncation of the CARD8 protein, which abolishes its ability to appropriately regulate the NF-κB signaling pathway (Fontalba et al. 2007). Concerning other neurological diseases, females with the *CARD8* rs2043211 TT genotype were found to have a 2.39-fold lower risk of developing AD than individuals with the *CARD8* rs2043211 AA genotype (Fontalba et al. 2007).

An important finding of the study was the effect of genetic variability in the *MIR146A* gene, which was associated with the age of onset of symptoms, as well as RHS and RULM scores. After adjusting for *SMN2* copy number, we observed that individuals carrying at least one *MIR146A* rs2910164 C allele tended to develop symptoms at a later age. Similarly, we found that individuals with at least one *MIR146A* rs2910164 C allele were associated with higher RHS scores, even after adjusting for age, *SMN2* copy number, and disease duration. Additionally, carriers of at least one polymorphic *MIR146A* rs2910164 C allele had increased RULM scores, with the results remaining nominally significant even after adjustment. The SNP *MIR146A* rs2910164 (C > G) is a common variation in *MIR146A* that has the potential to alter miRNA expression and activity (Wang et al. 2017). MiR-146a is an important regulator of acquired immunity and inflammatory responses and likely plays an important role in astrocyte-mediated inflammation. As a negative feedback regulator, it targets the *TRAF6* and *IRAK1*, which are downstream of Toll-like and cytokine receptors and are critical for proinflammatory signaling (Taganov et al. 2006). In contrast, miR-146a is critical for regulatory T-cell function. Decreased expression interferes with IFN-γ responses and the inhibitory function of T cells (Rusca & Monticelli 2011).

MiR-146a levels were found to be increased in SMA iPSC-derived astrocytes and SMNΔ7 mouse spinal cord cells. These alterations appeared to contribute to the SMA astrocyte-induced loss of MNs in vitro, which could be prevented by the inhibition of miR-146a, suggesting that altered miR-146a production by astrocytes may play a role in SMA pathology (Sison et al. 2017). When the data were compared between MS patients and control individuals, a highly significant difference was found between patients and controls in both the genotypic and allelic frequencies of *MIR146A* rs2910164 (Labib et al. 2018). In another study, a combination of the *MIR223* rs1044165 T allele and the *MIR146A* rs2910164 GG genotype was shown to correlate with an increased risk of MS in female patients of Russian ancestry (Kiselev et al. 2015). In contrast, another study revealed that the C allele of *MIR146A* rs2910164 is a risk factor for MS in the Iranian population (Shareef et al. 2021). Previous studies in AD patients also described the association of the rarer C allele with a higher risk of AD (Zhang et al. 2015) and the association of at least one polymorphic allele with a higher Aβ_42/40_ ratio (Vogrinc et al. 2023).

Polymorphisms in neurodevelopmental genes, particularly the *NOTCH* rs367398 A allele, were shown to be associated with increased RHS, RULM, VC (%), and PEF (%) scores, suggesting the potential impact of this SNP on the progression of motor and respiratory functions. The *NOTCH* polymorphism rs367398 involves an allelic substitution from A to G in the promoter region and can therefore influence transcription. Although the *NOTCH* polymorphism rs367398 has not yet been studied in the context of SMA, studies have described the role of the Notch signaling pathway in SMA. Specifically, SMN deficiency has been associated with increased expression of key Notch signaling pathway components (Caraballo-Miralles et al. 2013). Additionally, in a separate study, impaired Notch signaling was observed in a mouse model of SMA, and pharmacological inhibition effectively improved motor function deficits (Ohuchi et al. 2019). Concerning the role of *NOTCH* rs367398 in other neurodegenerative diseases, evidence of an increased risk of developing AD associated with the rs367398 CC genotype in ε4 carriers of the *APOE* gene was found in a British population (Lambert et al. 2004). However, this association was not confirmed either in the French population or in a follow-up study that examined a Japanese cohort (Shibata et al. 2007). Consistent with our findings, no significant association between the *NOTCH* rs367398 polymorphism and disease risk was observed in schizophrenia patients. However, the *NOTCH* rs367398 AA/AG variant was associated with higher scores on the Positive and Negative Syndrome Scale (PANSS) and the Clinical Global Impression (CGI) (Terzić et al. 2015).

Variations in the *HMOX1* and *HMOX2* genes, specifically the polymorphisms rs2071747, rs1051308, and rs2270363, were found to be associated with SMA in our study. Heme oxygenase (HMOX) catalyzes the breakdown of heme to biliverdin, which is subsequently converted to bilirubin. HMOX also produces iron ions and carbon monoxide, which are suspected neurotransmitters (Bonkovsky et al. 2013). There are two primary isoenzymes of HMOX: constitutive HMOX2 and inducible HMOX1. HMOX2 deficiency leads to the disruption of iron metabolism, resulting in iron deposition and the subsequent involvement of iron ions in redox reactions, which in turn results in the production of ROS and inflammatory factors (Kumar & Bandyopadhyay 2005). In addition, HMOX2 has been shown to have neuroprotective effects on cerebral hemorrhage injury (Zhang et al. 2021).

The *HMOX2* rs1051308 polymorphism is located in the 3'-untranslated region and represents a genetic variation in which the G nucleotide at position 544 is replaced by A. In our study, the *HMOX2* rs1051308 (AA) and *HMOX2* rs2270363 (GG) were associated with younger age at symptom onset. Analysis via a recessive model confirmed the association between the absence of the *HMOX2* rs1051308 A allele and late disease onset. In addition, we observed that carriers of the *HMOX2* rs1051308 A allele were more frequently associated with SMA type 2, a more severe form of the disease, compared to patients with SMA type 3. Comparable results were obtained in a case‒control association study that showed a weak association between the allelic variant *HMOX2* rs1051308A and MS risk in Spanish Caucasian men, suggesting that the A allele may contribute to the manifestation of the disease. However, the SNP studied was not associated with age at MS onset (Agúndez et al. 2016).

The *HMOX2* variant rs2270363, also known as c.-42 + 1444A > G, indicates a genetic alteration in which an A nucleotide is replaced by a G in the regulatory region of the human *HMOX2* gene. When examined for its effect on PD risk, carriers of the *HMOX2* rs2270363 GG genotype were found to have an increased risk of PD (Ayuso et al. 2011), consistent with our findings indicating the G allele as the risk allele. Another study revealed a possible link between the A-to-G transition in the *HMOX2* gene and the onset and progression of age-related macular degeneration (Synowiec et al. 2012). In contrast, a study in the Chinese population described an association between the risk of schizophrenia and the presence of the so-called risk allele A in *HMOX2* rs2270363. In addition, the authors observed concordance in the distribution of the risk allele of *HMOX2* rs2270363 in both the European and Chinese populations (Wang et al. 2022).

Our findings revealed that the *HMOX1* rs2071747 C allele was associated with lower RHS and VC (%) values. This polymorphism has not been described in SMA patients; however, there are a few reports of *HMOX1* rs2071747 in studies of other neurological disorders. Although there is evidence that the dominant model of *HMOX1* rs2071747 (GG/GC + CC) was significantly different between PD patients and controls after adjustment for age and sex (Xiong & Zhang 2022), most studies have reported no association between *HMOX1* rs2071747 and disease risk (Agúndez et al. 2017, 2016; Ayuso et al. 2015, 2014; García-Martín et al. 2015).

Given the importance of HMOX2 in oxidative stress-related diseases, the activation of cytoprotective enzymes, including HMOX2, by the KEAP1-NRF2-ARE pathway highlights the potential of this pathway as a therapeutic target for oxidative stress-related chronic diseases, such as SMA (Magesh et al. 2012). Indeed, our study demonstrated that genetic variations in *NFE2L2* rs6721961 and rs35652124 have an impact on the age of symptom onset, SMA type, and motor or lung function parameters in SMA patients. In particular, later symptom onset and increased PEF (%) were observed for *NFE2L2* rs35652124 C allele carriers. Additionally, individuals carrying at least one *NFE2L2* rs35652124 C allele were more likely to have SMA type 3, a milder form of the disease, compared to patients with SMA type 2. Similarly, carriers of the *NFE2L2* rs6721961 T allele had lower PEF (%) and RHS scores. Previous studies have suggested that the *NFE2L2* rs35652124 C allele plays a protective role against PD by delaying the onset of symptoms (Ran et al. 2017). Among AD patients, the presence of the polymorphic *NFE2L2* rs35652124 C allele was associated with lower cognitive test scores on the Mini-Mental State Examination (MMSE) (Vogrinc et al. 2023).

Regarding *NFE2L2* rs6721961, previous studies have reported that the T allele is linked to a reduced rate of cognitive decline and a decreased risk of PD (Paul et al. 2018). Interestingly, a splicing regulatory function related to the SMN protein has been described for NRF2, which is encoded by *NFE2L2*. Functionally, NRF2 regulates the transcription of SMN mRNA expression by binding to two antioxidant response elements within the *SMN1* promoter. In addition, NRF2 physically associates with the SMN protein at the posttranscriptional level (Cui et al. 2023). These findings are consistent with the findings of Nizzardo et al. (2011), who demonstrated the neuroprotective effect of beta-lactam antibiotics in an SMA model through several mechanisms, including NRF2 activation and increased SMN protein levels (Nizzardo et al. 2011).

The results of our study suggest that genetic variations in genes associated with oxidative stress, inflammation, and neurodevelopment have the potential to modify the disease course of SMA, influence patients' motor and respiratory functions, and provide further links between these factors and neurodegeneration. Several limitations of our study should be considered, including the lack of measurements of the mRNA levels of the studied genes. Second, the study had a modest sample size, although it included all eligible adult Slovenian patients, i.e., those with a genetically and clinically confirmed diagnosis of SMA for whom clinical data were available. Extending the scope of the study to encompass patients with different types of SMA, including SMA type 1, could significantly improve the results. This is particularly important given the challenges associated with identifying the effects of different factors in a study that is constrained by a limited sample size. Nevertheless, larger studies may face similar challenges due to inherent phenotypic heterogeneity within the population. Due to logistical challenges and other personal constraints, there were limitations in the collection of clinical parameters, resulting in some patients missing certain measurements, including RULM and RHS scores, as well as respiratory function parameters. Another limitation related to the clinical parameters was the presence of floor and ceiling effects. These effects could plausibly explain the observed similarities, though not identical results, in the analyses of associations between polymorphisms and the RHS or RULM scale scores, both of which evaluate patients' motor function.

Nonetheless, to the best of our knowledge, this study is the first comprehensive exploration providing evidence that polymorphisms in genes related to oxidative stress, inflammation, and neurodevelopmental pathways may contribute to the susceptibility to and progression of SMA. One notable strength of our study is the uniform recruitment of patients from a single center, where they underwent examinations following a standardized protocol. This approach enabled a comprehensive assessment of the collective influence of various clinical and genetic factors on the severity, course, and progression of SMA. Additionally, our pathway-based approach contributes to a more comprehensive understanding of disease mechanisms. Conducting a multicenter study with a larger sample size will be crucial for confirming our current findings and elucidating additional genotype‒phenotype correlations in the future. In addition, longitudinal studies capable of capturing changes in lung and motor function over time could provide a more comprehensive understanding of disease progression. We believe our findings have profound implications for future therapies and highlight the potential of recent genomic advances in customizing treatment approaches to improve outcomes for SMA patients.

## Supplementary Information

Below is the link to the electronic supplementary material.

Supplementary file1 (DOCX 122 KB)

## Data Availability

No datasets were generated or analysed during the current study.

## Abbreviations

AGTR1: Angiotensin II type 1 receptor
ACMG: American College of Medical Genetics and Genomics
BDNF: Brain-derived neurotrophic factor
CAT: Catalase
CARD8: Caspase recruitment domain-containing protein
CI: Confidence interval
CGI: Clinical Global Impression
ESS: Exonic splicing silencer
GPX: Glutathione peroxidase
GST: Glutathione S-transferase
HBB: β-Globin
HMOX: Heme oxygenase
HWE: Hardy‒Weinberg equilibrium
IL1B: Interleukin-1 beta
IL6: Interleukin-6
IL6R: Interleukin-6 receptor
KEAP1: Kelch-like ECH-associated protein 1
KASP: Competitive allele-specific polymerase chain reaction
MN: Motor neuron
MAF: Minor allele frequency
MMSE: Mini-Mental State Examination
MS: Multiple sclerosis
NFE2L2: Nuclear factor, erythroid 2-like 2
NLRP3: NLR family pyrin domain containing 3
NMECRS: National Medical Ethics Committee of the Republic of Slovenia
NOS1: Nitric oxide synthase 1
OR: Odds ratio
PANSS: Positive and Negative Syndrome Scale
PEF: Peak expiratory flow
ROS: Reactive oxygen species
RHS: Revised Hammersmith Scale
RULM: Revised Upper Limb Module
SMA: Spinal muscular atrophy
SMN: Survival motor neuron protein
*SMN1*: Survival of motor neuron 1 gene
*SMN2*: Survival of motor neuron 2 gene
SNP: Single nucleotide polymorphism
SOD: Superoxide dismutase
TNF: Tumor necrosis factor
VC: Vital capacity
