# Supplementary material for: Genetic Variability in Oxidative Stress, Inflammatory, and Neurodevelopmental Pathways: Impact on the Susceptibility and Course of Spinal Muscular Atrophy
Source: Cell Mol Neurobiol. 2024 Oct 27;44:71. doi: 10.1007/s10571-024-01508-y (PMC11513727; doi:10.1007/s10571-024-01508-y)
Supplement: Supplementary file 1 — Supplementary file1 (DOCX 122 KB) [file 10571_2024_1508_MOESM1_ESM.docx]

# Supplementary

**Table S1.** Genotype frequencies of investigated polymorphisms.

| **Gene** | **Polymorphism** | **Role** | **Genotype** | **All subjects (N = 217)**  **N (%)** | **MAF^a^ (%)** | **HWE *p*-value (control cohort)** |
| --- | --- | --- | --- | --- | --- | --- |
| *GSTP1* | rs1695 | p.Ile105Val | AA | 91 (41.9) | 0.36 | 0.827 |
|  |  |  | AG | 97 (44.7) |  |  |
|  |  |  | GG | 29  (13.4) |  |  |
|  | rs1138272 | p.Ala114Val | CC | 173 (79.7) | 0.11 | 0.992 |
|  |  |  | CT | 42 (19.4) |  |  |
|  |  |  | TT | 2 (0.9) |  |  |
| *SOD2* | rs4880 | p.Ala16Val | AA | 53 (24.4) | 0.48 | 0.832 |
|  |  |  | AG | 105 (48.4) |  |  |
|  |  |  | GG | 59 (27.2) |  |  |
| *CAT* | rs1001179 | c.-262C>T | CC | 112 (51.6) | 0.28 | 0.694 |
|  |  |  | CT | 87 (40.1) |  |  |
|  |  |  | TT | 18 (8.3) |  |  |
| *GPX1* | rs1050450 | p.Pro198Leu | GG | 111 (51.2) | 0.30 | 0.272 |
|  |  |  | GA | 85 (39.2) |  |  |
|  |  |  | AA | 21 (9.7) |  |  |
| *NFE2L2* | rs6706649 | c.-767C>T | CC | 173 (79.7) | 0.09 | 0.213 |
|  |  |  | CT | 44 (20.3) |  |  |
|  |  |  | TT | 0 |  |  |
|  | rs6721961 | c.-733T>G | GG | 161 (74.2) | 0.13 | 0.489 |
|  |  |  | GT | 51 (23.5) |  |  |
|  |  |  | TT | 5 (2.3) |  |  |
|  | rs35652124 | c.-769T>C | TT | 103 (47.5) | 0.31 | 0.457 |
|  |  |  | TC | 90 (41.5) |  |  |
|  |  |  | CC | 24 (11.1) |  |  |
| *KEAP1* | rs1048290 | p.Leu471= | GG | 83 (38.2) | 0.41 | 0.352 |
|  |  |  | GC | 95 (43.8) |  |  |
|  |  |  | CC | 39 (18) |  |  |
|  | rs9676881 | c.*548G>A | GG | 83 (38.2) | 0.42 | 0.136 |
|  |  |  | GA | 92 (42.4) |  |  |
|  |  |  | AA | 42 (19.4) |  |  |
| *HMOX1* | rs2071746 | c.-495A>T | AA | 66 (30.4) | 0.44 | 0.373 |
|  |  |  | AT | 112 (51.6) |  |  |
|  |  |  | TT | 39 (18) |  |  |
|  | rs2071747 | p.Asp7His | GG | 201 (92.6) | 0.03 | 0.686 |
|  |  |  | GC | 16 (7.4) |  |  |
|  |  |  | CC | 0 |  |  |
| *HMOX2* | rs1051308 | c.*544G>A | AA | 110 (50.7) | 0.28 | 0.785 |
|  |  |  | AG | 93 (42.9) |  |  |
|  |  |  | GG | 14 (6.5) |  |  |
|  | rs2270363 | c.-42+1444A>G | GG | 133 (61.3) | 0.24 | 0.059 |
|  |  |  | GA | 68 (31.3) |  |  |
|  |  |  | AA | 16 (7.4) |  |  |
| *IL1B* | rs1143623 | c.-1560G>C | CC | 120 (55.3) | 0.28 | 0.537 |
|  |  |  | CG | 80 (36.9) |  |  |
|  |  |  | GG | 17 (7.8) |  |  |
|  | rs16944 | c.-598T>C | GG | 100 (46.1) | 0.34 | 0.463 |
|  |  |  | GA | 91 (41.9) |  |  |
|  |  |  | AA | 26 (12) |  |  |
|  | rs1071676 | c.*505G>C | GG | 117 (53.9) | 0.26 | 0.588 |
|  |  |  | GC | 85 (39.2) |  |  |
|  |  |  | CC | 15 (6.9) |  |  |
| *IL6* | rs1800795 | c.-174G>C | GG | 75 (34.6) | 0.40 | 0.073 |
|  |  |  | GC | 96 (44.2) |  |  |
|  |  |  | CC | 46 (21.2) |  |  |
| *IL6R* | rs2228145 | p.Asp358Ala | AA | 86 (39.6) | 0.37 | 0.758 |
|  |  |  | AC | 100 (46.1) |  |  |
|  |  |  | CC | 31 (14.3) |  |  |
| *MIR146A* | rs2910164 | n.60G>C | GG | 129 (59.4) | 0.22 | 0.203 |
|  |  |  | GC | 73 (33.6) |  |  |
|  |  |  | CC | 15 (6.9) |  |  |
| *TNF* | rs1800629 | c.-308G>A | GG | 149 (68.7) | 0.19 | 0.379 |
|  |  |  | GA | 62 (28.6) |  |  |
|  |  |  | AA | 6 (2.8) |  |  |
| *NLRP3* | rs35829419 | p.Gln705Lys | CC | 199 (92.2) | 0.04 | 0.596 |
|  |  |  | CA | 17 (7.8) |  |  |
|  |  |  | AA | 0 |  |  |
| *NOS1* | rs2293054 | p.Ile734= | GG | 135 (62.2) | 0.22 | 0.737 |
|  |  |  | GA | 71 (32.7) |  |  |
|  |  |  | AA | 11 (5.1) |  |  |
|  | rs2682826 | c.*276G>A | GG | 122 (56.2) | 0.25 | 0.938 |
|  |  |  | GA | 79 (36.4) |  |  |
|  |  |  | AA | 16 (7.4) |  |  |
| *CARD8* | rs2043211 | p.Cys10Ter | AA | 94 (43.3) | 0.35 | 0.840 |
|  |  |  | AT | 100 (46.1) |  |  |
|  |  |  | TT | 23 (10.6) |  |  |
| *BDNF* | rs6265 | p.Val66Met | CC | 144 (66.4) | 0.21 | 0.956 |
|  |  |  | CT | 62 (28.6) |  |  |
|  |  |  | TT | 11 (5.1) |  |  |
|  | rs28722151 | c.-21-1044G>C | CC | 54 (24.9) | 0.50 | 0.308 |
|  |  |  | CG | 111 (51.2) |  |  |
|  |  |  | GG | 52 (24) |  |  |
|  | rs11030101 [1] | c.-21-612T>A | AA | 57 (26.3) | 0.49 | 0.306 |
|  |  |  | AT | 110 (50.7) |  |  |
|  |  |  | TT | 49 (22.6) |  |  |
| *NOTCH* | rs367398 | c.-25C>T | GG | 81 (37.3) | 0.37 | 0.482 |
|  |  |  | GA | 108 (49.8) |  |  |
|  |  |  | AA | 28 (12.9) |  |  |

^a^Polymorphic allele more common in the European population. SMA: spinal muscular atrophy; HWE: Hardy-Weinberg equilibrium; MAF: minor allele frequency. The number of missing data is presented in [] brackets.

**Table S2**. Comparison of genotype frequencies of all polymorphisms between SMA patients (N = 54) and controls (N = 163).

| **Gene** | **Polymorphism** | **Genotype** | **Controls**  **N (%)** | **SMA**  **N (%)** | **OR (95% CI)** | ***p*-value** |
| --- | --- | --- | --- | --- | --- | --- |
| *GSTP1* | rs1695 | AA | 67 (41.1) | 24 (44.4) | Reference |  |
|  |  | AG | 74 (45.7) | 23 (42.6) | 0.868 (0.448–1.680) | 0.674 |
|  |  | GG | 22 (13.5) | 7 (13) | 0.888 (0.337–2.343) | 0.811 |
|  |  | AG+GG | 96 (58.9) | 30 (55.6) | 0.872 (0.469–1.623) | 0.667 |
|  | rs1138272 | CC | 129 (79.1) | 44 (81.5) | Reference |  |
|  |  | CT | 32 (19.8) | 10 (18.5) | 0.916 (0.417–2.015) | 0.828 |
|  |  | TT | 2 (1.2) | 0 | 0 | 1.000** |
|  |  | CT+TT | 34 (20.9) | 10 (18.5) | 0.862 (0.394–1.888) | 0.711 |
| *SOD2* | rs4880 | AA | 38 (23.3) | 15 (27.8) | Reference |  |
|  |  | AG | 80 (49.1) | 25 (46.3) | 0.792 (0.375–1.672) | 0.540 |
|  |  | GG | 45 (27.6) | 14 (25.9) | 0.788 (0.338–1.838) | 0.582 |
|  |  | AG+GG | 125 (76.7) | 39 (72.2) | 0.790 (0.394–1.588) | 0.509 |
| *CAT* | rs1001179 | CC | 85 (52.1) | 27 (50) | Reference |  |
|  |  | CT | 64 (39.3) | 23 (42.6) | 1.131 (0.594–2.154) | 0.707 |
|  |  | TT | 14 (8.6) | 4 (7.4) | 0.899 (0.273–2.964) | 0.862 |
|  |  | CT+TT | 78 (47.9) | 27 (50) | 1.090 (0.589–2.017) | 0.784 |
| *GPX1* | rs1050450 | GG | 82 (50.3) | 29 (53.7) | Reference |  |
|  |  | GA | 63 (38.7) | 22 (40.7) | 0.987 (0.519–1.880) | 0.969 |
|  |  | AA | 18 (11.0) | 3 (5.6) | 0.471 (0.129–1.718) | 0.254 |
|  |  | GA+AA | 81 (49.7) | 25 (46.3) | 0.873 (0.471–1.617) | 0.665 |
| *NFE2L2* | rs6706649 | CC | 134 (82.2) | 39 (72.2) | Reference |  |
|  |  | CT+TT | 29 (17.8) | 15 (27.8) | 1.777 (0.867–3.644) | 0.117 |
|  | *rs6721961 | GG | 123 (75.5) | 38 (70.4) | Reference |  |
|  |  | GT | 36 (22.1) | 15 (27.8) | 1.349 (0.667–2.726) | 0.405 |
|  |  | TT | 4 (2.5) | 1 (1.9) | 0.809 (0.088–7.460) | 0.852 |
|  |  | GT+TT | 40 (24.5) | 16 (29.6) | 1.295 (0653–2.567) | 0.459 |
|  | rs35652124 | TT | 79 (48.5) | 24 (44.4) | Reference |  |
|  |  | TC | 66 (40.5) | 24 (44.4) | 1.197 (0.623–2.301) | 0.590 |
|  |  | CC | 18 (11.0) | 6 (11.1) | 1.097 (0.391–3.076) | 0.860 |
|  |  | TC+CC | 84 (51.5) | 30 (55.6) | 1.176 (0.633–2.182) | 0.608 |
| *KEAP1* | rs1048290 | GG | 60 (36.8) | 23 (42.6) | Reference |  |
|  |  | GC | 73 (44.8) | 22 (40.7) | 0.786 (0.400–1.547) | 0.486 |
|  |  | CC | 30 (18.4) | 9 (16.7) | 0.783 (0.323–1.899) | 0.588 |
|  |  | GC+CC | 103 (63.2) | 31 (57.4) | 0.785 (0.420–1.469) | 0.449 |
|  | rs9676881 | GG | 60 (36.8) | 23 (42.6) | Reference |  |
|  |  | GA | 70 (42.9) | 22 (40.7) | 0.820 (0.416–1.616) | 0.566 |
|  |  | AA | 33 (20.2) | 9 (16.7) | 0.711(0.295–1.715) | 0.448 |
|  |  | GA+AA | 103 (63.2) | 31 (57.4) | 0.785 (0.420–1.469) | 0.449 |
| *HMOX1* | rs2071746 | AA | 48 (29.4) | 18 (33.3) | Reference |  |
|  |  | AT | 86 (52.9) | 26 (48.1) | 0.806 (0.402–1.619) | 0.545 |
|  |  | TT | 29 (17.8) | 10 (18.5) | 0.920 (0.374–2.262) | 0.855 |
|  |  | AT+TT | 115 (70.6) | 36 (66.7) | 0.835 (0.432–1.613) | 0.591 |
|  | rs2071747 | GG | 153 (93.9) | 48 (88.9) | Reference |  |
|  |  | GC+CC | 10 (6.1) | 6 (11.1) | 1.912 (0.661–5.536) | 0.232 |
| *HMOX2* | *rs1051308 | AA | 84 (51.5) | 26 (48.1) | Reference |  |
|  |  | AG | 67 (41.1) | 26 (48.1) | 1.254 (0.667–2.357) | 0.483 |
|  |  | GG | 12 (7.4) | 2 (3.7) | 0.538 (0.113–2.563) | 0.437 |
|  |  | AG+GG | 79 (48.5) | 28 (51.9) | 1.145 (0.619–2.120) | 0.666 |
|  | *rs2270363 | GG | 98 (60.1) | 35 (64.8) | Reference |  |
|  |  | GA | 51 (31.3) | 17 (31.5) | 0.933 (0.477–1.826) | 0.840 |
|  |  | AA | 14 (8.6) | 2 (3.7) | 0.400 (0.087–1.849) | 0.241 |
|  |  | GA+AA | 65 (39.9) | 19 (35.2) | 0.818 (0.431–1.553) | 0.540 |
| *IL1B* | rs1143623 | CC | 87 (53.4) | 33 (61.1) | Reference |  |
|  |  | CG | 62 (38) | 18 (33.3) | 0.765 (0.395–1.481) | 0.427 |
|  |  | GG | 14 (8.6) | 3 (5.6) | 0.565 (0.152–2.093) | 0.393 |
|  |  | CG+GG | 76 (46.6) | 21 (38.9) | 0.728 (0.389–1.365) | 0.323 |
|  | *rs16944 | GG | 73 (44.8) | 27 (50) | Reference |  |
|  |  | GA | 69 (42.3) | 22 (40.7) | 0.862 (0.449–1.655) | 0.655 |
|  |  | AA | 21 (12.9) | 5 (9.3) | 0.644 (0.221–1.878) | 0.420 |
|  |  | GA+AA | 90 (55.2) | 27 (50) | 0.811 (0.438–1.502) | 0.506 |
|  | rs1071676 | CC | 87 (53.4) | 30 (55.6) | Reference |  |
|  |  | CG | 66 (40.5) | 19 (35.2) | 0.835 (0.432–1.612) | 0.591 |
|  |  | GG | 10 (6.1) | 5 (9.3) | 1.450 (0.459–4.584) | 0.527 |
|  |  | CG+GG | 76 (46.6) | 24 (44.4) | 0.916 (0.493–1.700) | 0.781 |
| *IL6* | *rs1800795 | GG | 65 (39.9) | 10 (18.5) | Reference |  |
|  |  | GC | 67 (41.1) | 29 (39.2) | 2.813 (1.270–6.234) | **0.011** |
|  |  | CC | 31 (19.0) | 15 (27.8) | 3.145 (1.269–7.793) | **0.013** |
|  |  | GC+CC | 98 (60.1) | 44 (81.5) | 2.918 (1.372–6.208) | **0.005** |
| *IL6R* | rs2228145 | AA | 66 (40.5) | 20 (37) | Reference |  |
|  |  | AC | 74 (45.4) | 26 (48.1) | 1.159 (0.593–2.268) | 0.666 |
|  |  | CC | 23 (14.1) | 8 (14.8) | 1.148 (0.445–2.960) | 0.775 |
|  |  | AC+CC | 97 (59.5) | 34 (63) | 1.157 (0.613–2.182) | 0.653 |
| *MIR146A* | *rs2910164 | GG | 101 (62) | 28 (51.9) | Reference |  |
|  |  | GC | 51 (31.3) | 22 (40.7) | 1.556 (0.811–2.987) | 0.184 |
|  |  | CC | 11 (6.7) | 4 (7.4) | 1.312 (0.388–4.436) | 0.663 |
|  |  | GC+CC | 62 (38) | 26 (48.1) | 1.513 (0.813–2.813) | 0.191 |
| *TNF* | rs1800629 | GG | 106 (65) | 43 (79.6) | Reference |  |
|  |  | GA | 53 (32.5) | 9 (16.7) | 0.419 (0.190–0.923) | **0.031** |
|  |  | AA | 4 (2.5) | 2 (3.7) | 1.233 (0.218–6.980) | 0.813 |
|  |  | GA+AA | 57 (35) | 11 (20.4) | 0.476 (0.228–0.993) | **0.048** |
| *NLRP3* | rs35829419 | CC | 150 (92) | 50 (92.6) | Reference |  |
|  |  | CA | 13 (8) | 4 (7.4) | 0.923 (0.288–2.961) | 0.893 |
| *NOS1* | *rs2293054 | GG | 99 (60.7) | 36 (66.7) | Reference |  |
|  |  | GA | 57 (35) | 14 (25.9) | 0.675 (0.336–1.358) | 0.271 |
|  |  | AA | 7 (4.3) | 4 (7.4) | 1.571 (0.434–5.688) | 0.491 |
|  |  | GA+AA | 64 (39.3) | 18 (33.3) | 0.773 (0.405–1.477) | 0.437 |
|  | rs2682826 | GG | 93 (57.1) | 29 (53.7) | Reference |  |
|  |  | GA | 60 (36.8) | 19 (35.2) | 1.052 (0.540–2.049) | 0.882 |
|  |  | AA | 10 (6.1) | 6 (11.1) | 1.993 (0.666–5.968) | 0.218 |
|  |  | GA+AA | 70 (42.9) | 25 (46.3) | 1.145 (0.617–2.125) | 0.667 |
| *CARD8* | rs2043211 | AA | 69 (42.3) | 25 (46.3) | Reference |  |
|  |  | AT | 75 (46) | 25 (46.3) | 0.920 (0.483–1.751) | 0.800 |
|  |  | TT | 19 (11.7) | 4 (7.4) | 0.581 (0.180–1.875) | 0.364 |
|  |  | AT+TT | 94 (57.7) | 29 (53.7) | 0.851 (0.459–1.581) | 0.610 |
| *BDNF* | rs6265 | CC | 103 (63.2) | 41 (75.9) | Reference |  |
|  |  | CT | 53 (32.5) | 9 (16.7) | 0.427 (0.193–0.944) | **0.035** |
|  |  | TT | 7 (4.3) | 4 (7.4) | 1.436 (0.399–5.167) | 0.580 |
|  |  | CT+TT | 60 (36.8) | 13 (24.1) | 0.544 (0.270–1.097) | 0.089 |
|  | rs28722151 | CC | 38 (23.3) | 16 (29.6) | Reference |  |
|  |  | CG | 88 (54) | 23 (42.6) | 0.621 (0.295–1.305) | 0.208 |
|  |  | GG | 37 (22.7) | 15 (27.8) | 0.963 (0.417–2.224) | 0.929 |
|  |  | CG+GG | 125 (76.7) | 38 (70.4) | 0.722 (0.363–1.436) | 0.353 |
|  | rs11030101 [1] | AA | 39 (23.9) | 18 (33.3) | Reference |  |
|  |  | AT | 88 (54) | 22 (40.7) | 0.542 (0.262–1.122) | 0.099 |
|  |  | TT | 36 (22.1) | 13 (24.1) | 0.782 (0.336–1.822) | 0.569 |
|  |  | AT+TT | 124 (76.1) | 35 (66) | 0.612 (0.312–1.198) | 0.152 |
| *NOTCH* | rs367398 | GG | 63 (38.7) | 18 (33.3) | Reference |  |
|  |  | GA | 80 (49.1) | 28 (51.9) | 1.225 (0.622–2.413) | 0.557 |
|  |  | AA | 20 (12.3) | 8 (14.8) | 1.400 (0.529–3.704) | 0.498 |
|  |  | GA+AA | 100 (61.3) | 36 (66.7) | 1.260 (0.659–2.408) | 0.484 |
| *GSTM1*-present | | | 67 (41.1) | 22 (40.7) | Reference |  |
| *GSTM1*-null | | | 96 (58.9) | 32 (59.3) | 1.015 (0.543–1.899) | 0.962 |
| *GSTT1*-present | | | 146 (89.6) | 44 (81.5) | Reference |  |
| *GSTT1*-null | | | 17 (10.4) | 10 (18.5) | 1.952 (0.834–4.570) | 0.123 |

*A recessive model was used. **Calculated using Fisher’s exact test. Homozygotes for wild-type alleles matched with the dbSNP were used as reference, except for *NFE2L2* rs6721961, *HMOX2 rs*1051308, rs2270363, *IL6* rs1800795, *MIR146A* rs2910164, *NOS1* rs2293054. SMA: Spinal muscular atrophy; CI: confidence interval; OR: odds ratio. The number of missing data is presented in [] brackets. Nominally significant results are printed in bold text.

**Table S3.** Comparison of genotype frequencies of all polymorphisms among patients with SMA types 2 and 3 (N = 44).

| **Gene** | **Polymorphism** | **Genotype** | **SMA Type 2 (N = 21)** | **SMA Type 3 (N = 23)** | **OR (95% CI)** | ***p*-*value*** | **OR_adj_ (95% CI)** | ***p*_adj_-value** |
| --- | --- | --- | --- | --- | --- | --- | --- | --- |
| *GSTP1* | rs1695 | AA | 7 (33.3) | 13 (56.5) | Reference |  |  |  |
|  |  | AG | 10 (47.6) | 8 (34.8) | 0.431 (0.117–1.592) | 0.207 | 0.666 (0.153–2.895) | 0.588 |
|  |  | GG | 4 (19.0) | 2 (8.7) | 0.269 (0.039–1.855) | 0.183 | 0.551 (0.066– 4.615) | 0.583 |
|  |  | AG+GG | 14 (66.7) | 10 (43.5) | 0.385 (0.113–1.310) | 0.127 | 0.638 (0.159–2.556) | 0.525 |
|  | rs1138272 | CC | 14 (66.7) | 21 (91.3) | Reference |  |  |  |
|  |  | CT+TT | 7 (33.3) | 2 (8.7) | 0.2190 (0.034–1.054) | 0.057 | 0.250 (0.039–1.602) | 0.144 |
| *SOD2* | rs4880 | AA | 6 (28.6) | 5 (21.7) | Reference |  |  |  |
|  |  | AG | 9 (42.9) | 12 (52.2) | 1.600 (0.369–6.946) | 0.530 | 0.995 (0.171–5.777) | 0.995 |
|  |  | GG | 6 (28.6) | 6 (26.1) | 1.200 (0.233–6.185) | 0.827 | 1.207 (0.188– 7.741) | 0.843 |
|  |  | AG+GG | 15 (71.4) | 18 (78.3) | 1.440 (0.366–5.669) | 0.602 | 1.081 (0.217– 5.387) | 0.925 |
| *CAT* | rs1001179 | CC | 11 (52.4) | 10 (43.5) | Reference |  |  |  |
|  |  | CT | 9 (42.9) | 11 (47.8) | 1.344 (0.394–4.593) | 0.637 | 0.909 (0.213–3.882) | 0.897 |
|  |  | TT | 1 (4.8) | 2 (8.7) | 2.200 (0.172–28.14) | 0.544 | 2.710 (0.172–42.68) | 0.478 |
|  |  | CT+TT | 10 (47.6) | 13 (56.5) | 1.430 (0.436–4.694) | 0.555 | 1.075 (0.273–4.233) | 0.918 |
| *GPX1* | rs1050450 | GG | 13 (61.9) | 9 (39.1) | Reference |  |  |  |
|  |  | GA | 7 (33.3) | 12 (52.2) | 2.476 (0.701–8.742) | 0.159 | 2.952 (0.687–12.69) | 0.146 |
|  |  | AA | 1 (4.8) | 2 (8.7) | 2.889 (0.226–36.87) | 0.414 | 4.759 (0.290–78.04) | 0.274 |
|  |  | GA+AA | 8 (38.1) | 14 (60.9) | 2.528 (0.750–8.522) | 0.135 | 3.154 (0.771–12.90) | 0.110 |
| *NFE2L2* | rs6706649 | CC | 18 (85.7) | 15 (65.2) | Reference |  |  |  |
|  |  | CT+TT | 3 (14.3) | 8 (34.8) | 3.200 (0.719–14.247) | 0.127 | 3.121 (0.601–16.20) | 0.176 |
|  | *rs6721961 | GG | 12 (57.1) | 17 (73.9) | Reference |  |  |  |
|  |  | GT | 8 (38.1) | 6 (26.1) | 0.529 (0.146–1.925) | 0.334 | 1.000 (0.228–4.388) | 1.000 |
|  |  | TT | 1 (4.8) | 0 | / | 0.419** | / | / |
|  |  | GT+TT | 9 (42.9) | 6 (26.1) | 0.471 (0.132–1.676) | 0.245 | 0.908 (0.211–3.910) | 0.897 |
|  | rs35652124 | TT | 13 (61.9) | 7 (30.4) | Reference |  |  |  |
|  |  | TC | 6 (28.6) | 12 (52.2) | 3.714 (0.969–14.23) | 0.056 | 2.569 (0.589–11.19) | 0.209 |
|  |  | CC | 2 (9.5) | 4 (17.4) | 3.714 (0.539–25.59) | 0.183 | 1.654 (0.177–15.50) | 0.659 |
|  |  | TC+CC | 8 (38.1) | 16 (69.6) | 3.714 (1.063–12.98) | **0.040** | 2.336 (0.586–9.319) | 0.229 |
| *KEAP1* | rs1048290 | GG | 11 (52.4) | 8 (34.8) | Reference |  |  |  |
|  |  | GC | 7 (33.3) | 10 (43.5) | 1.964 (0.521–7.409) | 0.319 | 2.430 (0.483–12.22) | 0.281 |
|  |  | CC | 3 (14.3) | 5 (21.7) | 2.292 (0.420–12.50) | 0.338 | 5.189 (0.695–38.73) | 0.108 |
|  |  | GC+CC | 10 (47.6) | 15 (65.2) | 2.062 (0.614–6.932) | 0.242 | 3.100 (0.698–13.78) | 0.137 |
|  | rs9676881 | GG | 11 (52.4) | 8 (34.8) | Reference |  |  |  |
|  |  | GA | 7 (33.3) | 10 (43.5) | 1.964 (0.521–7.409) | 0.319 | 2.430 (0.483–12.22) | 0.281 |
|  |  | AA | 3 (14.3) | 5 (21.7) | 2.292 (0.420–12.50) | 0.338 | 5.189 (0.695–38.73) | 0.108 |
|  |  | GA+AA | 10 (47.6) | 15 (65.2) | 2.062 (0.614–6.932) | 0.242 | 3.100 (0.698–13.78) | 0.137 |
| *HMOX1* | rs2071746 | AA | 6 (28.6) | 9 (39.1) | Reference |  |  |  |
|  |  | AT | 12 (57.1) | 9 (39.1) | 0.500 (0.130–1.923) | 0.313 | 0.190 (0.031–1.171) | 0.073 |
|  |  | TT | 3 (14.3) | 5 (21.7) | 0.907 (0.190–6.492) | 0.907 | 1.030 (0.141–7.531) | 0.977 |
|  |  | AT+TT | 15 (71.4) | 14 (60.9) | 0.622 (0.176–2.202) | 0.462 | 0.354 (0.079–1.592) | 0.176 |
|  | rs2071747 | GG | 20 (95.2) | 18 (78.3) | Reference |  |  |  |
|  |  | GC+CC | 1 (4.8) | 5 (21.7) | 5.556 (0.592–52.16) | 0.133 | 3.896 (0.331–45.83) | 0.280 |
| *HMOX2* | *rs1051308 | AA | 15 (71.4) | 8 (34.8) | Reference |  |  |  |
|  |  | AG | 6 (28.6) | 14 (60.9) | 4.375 (1.210–15.81) | **0.024** | 3.411 (0.836–13.92) | 0.087 |
|  |  | GG | 0 | 1 (4.3) | / | **0.033**** | / | / |
|  |  | AG+GG | 6 (28.6) | 15 (65.2) | 4.687 (1.306–16.82) | **0.018** | 3.552 (0.873–14.45) | 0.077 |
|  | *rs2270363 | GG | 15 (71.4) | 14 (60.9) | Reference |  |  |  |
|  |  | GA | 6 (28.6) | 8 (34.8) | 1.429 (0.395–5.163) | 0.586 | 0.853 (0.191–3.820) | 0.836 |
|  |  | AA | 0 | 1 (4.3) | / | 0.868** | / | / |
|  |  | GA+AA | 6 (28.6) | 9 (39.1) | 1.607 (0.454–5.688) | 0.462 | 0.922 (0.209–4.061) | 0.915 |
| *IL1B* | rs1143623 | CC | 13 (61.9) | 14 (60.9) | Reference |  |  |  |
|  |  | CG | 6 (28.6) | 8 (34.8) | 1.238 (0.337–4.543) | 0.747 | 0.878 (0.190–4.047) | 0.867 |
|  |  | GG | 2 (9.5) | 1 (4.3) | 0.464 (0.037–5.749) | 0.550 | 0.179 (0.010–3.154) | 0.240 |
|  |  | CG+GG | 8 (38.1) | 9 (39.1) | 1.045 (0.310–3.522) | 0.944 | 0.665 (0.159–2.783) | 0.577 |
|  | *rs16944 | GG | 10 (47.6) | 12 (52.2) | Reference |  |  |  |
|  |  | GA | 8 (38.1) | 10 (43.5) | 1.042 (0.298–3.645) | 0.949 | 0.698 (0.160–3.047) | 0.632 |
|  |  | AA | 3 (14.3) | 1 (4.3) | 0.278 (0.025–3.104) | 0.298 | 0.133 (0.008–2.147) | 0.155 |
|  |  | GA+AA | 11 (52.4) | 11 (47.8) | 0.833 (0.255–2.724) | 0.763 | 0.531 (0.131–2.151) | 0.375 |
|  | rs1071676 | CC | 12 (57.1) | 14 (60.9) | Reference |  |  |  |
|  |  | CG | 6 (28.6) | 7 (30.4) | 1.000 (0.263–3.802) | 1.000 | 0.685 (0.143–3.266) | 0.635 |
|  |  | GG | 3 (14.3) | 2 (8.7) | 0.571 (0.081–4.009) | 0.573 | 0.839 (0.099–7.080) | 0.872 |
|  |  | CG+GG | 9 (42.9) | 9 (39.1) | 0.857 (0.257–2.856) | 0.802 | 0.729 (0.184–2.891) | 0.653 |
| *IL6* | *rs1800795 | GG | 3 (14.3) | 5 (21.7) | Reference |  |  |  |
|  |  | GC | 12 (57.1) | 10 (43.5) | 0.500 (0.095–2.628) | 0.413 | 0.699 (0.106–4.615) | 0.710 |
|  |  | CC | 6 (28.6) | 8 (34.8) | 0.800 (0.135–4.745) | 0.806 | 1.348 (0.169–10.72) | 0.778 |
|  |  | GC+CC | 18 (85.7) | 18 (78.3) | 0.600 (0.124–2.894) | 0.525 | 0.882 (0.147–5.288) | 0.890 |
| *IL6R* | rs2228145 | AA | 10 (47.6) | 6 (26.1) | Reference |  |  |  |
|  |  | AC | 6 (28.6) | 14 (60.9) | 3.889 (0.966–15.65) | 0.056 | 4.782 (0.926–24.70) | 0.062 |
|  |  | CC | 5 (23.8) | 3 (13.0) | 1.000 (0.173–5.772) | 1.000 | 1.643 (0.217–12.42) | 0.631 |
|  |  | AC+CC | 11 (52.4) | 17 (73.9) | 2.576 (0.727–9.124) | 0.143 | 3.463 (0.756–15.86) | 0.110 |
| *MIR146A* | *rs2910164 | GG | 15 (71.4) | 11 (47.8) | Reference |  |  |  |
|  |  | GC | 5 (23.8) | 11 (47.8) | 3.000 (0.807–11.15) | 0.101 | 3.875 (0.839–17.90) | 0.083 |
|  |  | CC | 1 (4.8) | 1 (4.3) | 1.364 (0.077–24.27) | 0.833 | 0.364 (0.017–7.901) | 0.520 |
|  |  | GC+CC | 6 (28.6) | 12 (52.2) | 2.727 (0.780–9.531) | 0.116 | 2.853 (0.699–11.64) | 0.144 |
| *TNF* | rs1800629 | GG | 16 (77.2) | 19 (82.6) | Reference |  |  |  |
|  |  | GA | 3 (14.3) | 4 (17.4) | 1.123 (0.218–5.777) | 0.890 | 0.872 (0.138–5.506) | 0.884 |
|  |  | AA | 2 (9.5) | 0 | / | 0.448** | / | / |
|  |  | GA+AA | 5 (23.8) | 4 (17.4) | 0.674 (0.154–2.940) | 0.599 | 0.600 (0.112–3.219) | 0.551 |
| *NLRP3* | rs35829419 | CC | 21 (100) | 18 (82.6) | Reference |  |  |  |
|  |  | CA+AA | 0 | 4 (17.4) | / | 0.109** | / | / |
| *NOS1* | *rs2293054 | GG | 15 (71.4) | 15 (65.2) | Reference |  |  |  |
|  |  | GA | 3 (14.3) | 8 (34.8) | 2.667 (0.591–12.04) | 0.202 | 4.778 (0.846–26.98) | 0.077 |
|  |  | AA | 3 (14.3) | 0 | / | 0.752** | / | / |
|  |  | GA+AA | 6 (28.6) | 8 (34.8) | 1.333 (0.372–4.785) | 0.659 | 2.606 (0.565–12.02) | 0.219 |
|  | rs2682826 | GG | 10 (47.6) | 14 (60.9) | Reference |  |  |  |
|  |  | GA | 8 (38.1) | 7 (30.4) | 0.625 (0.170–2.291) | 0.478 | 1.099 (0.247–4.894) | 0.902 |
|  |  | AA | 3 (14.3) | 2 (8.7) | 0.476 (0.067–3.396) | 0.459 | 0.571 (0.062–5.276) | 0.621 |
|  |  | GA+AA | 11 (52.4) | 9 (39.1) | 0.379 (0.176–1.936) | 0.379 | 0.931 (0.236–3.665) | 0.918 |
| *CARD8* | rs2043211 | AA | 15 (71.4) | 6 (26.1) | Reference |  |  |  |
|  |  | AT | 4 (19.0) | 15 (65.2) | 9.375 (2.191–40.11) | **0.003** | 7.639 (1.563–37.35) | **0.012** |
|  |  | TT | 2 (9.5) | 2 (8.7) | 2.500 (0.284–22.04) | 0.409 | 1.901 (0.169–21.34) | 0.603 |
|  |  | AT+TT | 6 (28.6) | 17 (73.9) | 7.083 (1.878–26.72) | **0.004** | 5.669 (1.328–24.19) | **0.019** |
| *BDNF* | rs6265 | CC | 15 (71.4) | 21 (91.3) | Reference |  |  |  |
|  |  | CT | 4 (19.0) | 2 (8.7) | 0.357 (0.058–2.209) | 0.268 | 0.184 (0.019–1.794) | 0.145 |
|  |  | TT | 2 (9.5) | 0 | / | 0.126** | / | / |
|  |  | CT+TT | 6 (28.6) | 2 (8.7) | 0.238 (0.042–1.346) | 0.104 | 0.112 (0.013–0.974) | **0.047** |
|  | rs28722151 | CC | 5 (23.8) | 9 (39.1) | Reference |  |  |  |
|  |  | CG | 12 (57.1) | 8 (34.8) | 0.370 (0.090–1.521) | 0.168 | 0.218 (0.038–1.239) | 0.086 |
|  |  | GG | 4 (19.0) | 6 (26.1) | 0.833 (0.157–4.436) | 0.831 | 0.573 (0.082–3.999) | 0.574 |
|  |  | CG+GG | 16 (76.2) | 14 (60.9) | 0.486 (0.131–1.797) | 0.280 | 0.307 (0.064–1.478) | 0.141 |
|  | rs11030101 [1] | AA | 6 (28.6) | 10 (45.5) | Reference |  |  |  |
|  |  | AT | 11 (52.4) | 7 (31.8) | 0.382 (0.095–1.528) | 0.174 | 0.249 (0.046–1.340) | 0.105 |
|  |  | TT | 4 (19.0) | 5 (22.7) | 0.750 (0.143–3.941) | 0.734 | 0.610 (0.091–4.091) | 0.611 |
|  |  | AT+TT | 15 (71.4) | 12 (54.5) | 0.480 (0.135–1.701) | 0.256 | 0.344 (0.077–1.541) | 0.163 |
| *NOTCH* | rs367398 | GG | 8 (38.1) | 8 (34.8) | Reference |  |  |  |
|  |  | GA | 10 (47.6) | 12 (52.2) | 1.200 (0.330–4.360) | 0.782 | 1.174 (0.263–5.249) | 0.834 |
|  |  | AA | 3 (14.3) | 3 (13.0) | 1.000 (0.153–6.531) | 1.000 | 1.977 (0.245–15.94) | 0.522 |
|  |  | GA+AA | 13 (61.9) | 15 (65.2) | 1.154 (0.337–3.946) | 0.820 | 1.326 (0.323–5.434) | 0.695 |
| *GSTM1*-present | | | 10 (47.6) | 9 (39.1) | Reference |  |  |  |
| *GSTM1*-null | | | 11 (52.4) | 14 (60.9) | 1.414 (0.427–4.685) | 0.571 | 1.424 (0.366–5.547) | 0.610 |
| *GSTT1*-present | | | 16 (76.2) | 21 (91.3) | Reference |  |  |  |
| *GSTT1*-null | | | 5 (23.8) | 2 (8.7) | 0.305 (0.052–1.779) | 0.187 | 0.533 (0.078–3.617) | 0.519 |

*A recessive model was used. **Calculated using Fisher’s exact test. Homozygotes for wild-type alleles matched with the dbSNP were used as reference, except for *NFE2L2* rs6721961, *HMOX2 rs*1051308, rs2270363, *IL6* rs1800795, *MIR146A* rs2910164, *NOS1* rs2293054. Adj: adjusted for *SMN2* copy number; SMA: Spinal muscular atrophy. The number of missing data is presented in [] brackets. Nominally significant results are printed in bold text.

**Table S4.** Association of investigated genetic variant with age of symptom onset (N = 46).

| **Gene** | **Polymorphism** | **Genotype** | **SMA**  **N (%)** | **Age at symptom onset, Median (25**–**75%)** | ***p*-value** | ***p*_adj_-value** |
| --- | --- | --- | --- | --- | --- | --- |
| *GSTP1* | rs1695 | AA | 21 (45.7) | 6.00 (2.50–14.50) | 0.113 | 0.883 |
|  |  | AG | 19 (41.3) | 1.00 (1.50–10.00) |  |  |
|  |  | GG | 6 (13.0) | 0.65 (1.65–3.25) |  |  |
|  |  | AG+GG | 25 (54.3) | 1.50 (1.00–6.00) | 0.057 | 0.645 |
|  | rs1138272 | CC | 37 (80.4) | 4.00 (1.13–12.00) | 0.217 | 0.554 |
|  |  | CT+TT | 9 (16.6) | 1.00 (1.30–3.00) |  |  |
| *SOD2* | rs4880 | AA | 11 (23.9) | 3.00 (0.90–16.00) | 0.964 | 0.067 |
|  |  | AG | 23 (50.0) | 3.00 (1.00–12.00) |  |  |
|  |  | GG | 12 (26.1) | 3.00 (1.08–7.00) |  |  |
|  |  | AG+GG | 35 (76.1) | 3.00 (1.00–10.00) | 0.800 | 0.021 |
| *CAT* | rs1001179 | CC | 22 (47.8) | 3.00 (0.94–13.00) | 0.944 | **0.014** |
|  |  | CT | 20 (43.5) | 3.00 (1.26–10.00) |  |  |
|  |  | TT | 4 (8.7) | 5.30 (0.53–31.00) |  |  |
|  |  | CT+TT | 24 (52.2) | 3.00 (1.06–10.00) | 0.851 | 0.568 |
| *GPX1* | rs1050450 | GG | 24 (52.2) | 3.00 (1.13–14.00) | 0.891 | 0.495 |
|  |  | GA | 19 (41.3) | 4.00 (1.15–10.00) |  |  |
|  |  | AA | 3 (6.5) | 3.00 (2.00–5.00) |  |  |
|  |  | GA+AA | 22 (47.8) | 4.00 (1.00–10.00) | 0.741 | 0.244 |
| *NFE2L2* | rs6706649 | CC | 34 (67.4) | 3.00 (1.16–10.00) | 0.590 | 0.154 |
|  |  | CT+TT | 12 (26.1) | 3.00 (1.00–19.00) |  |  |
|  | *rs6721961 | GG | 31 (67.4) | 4.00 (1.30–14.00) | 0.146 | 0.868 |
|  |  | GT | 14 (30.4) | 2.50 (0.88–4.00) |  |  |
|  |  | TT | 1 (2.2) | / |  |  |
|  |  | GT+TT | 15 (32.6) | 2.00 (0.70–4.00) | 0.080 |  |
|  | rs35652124 | TT | 20 (43.5) | 1.75 (1.00–3.75) | 0.128 | 0.420 |
|  |  | TC | 20 (43.5) | 6.00 (0.99–15.50) |  |  |
|  |  | CC | 6 (13.0) | 8.00 (1.30–15.25) |  |  |
|  |  | TC+CC | 26 (56.5) | 7.00 (1.29–15.25) | **0.047** | 0.563 |
| *KEAP1* | rs1048290 | GG | 19 (41.3) | 3.00 (1.30–8.00) | 0.976 | 0.180 |
|  |  | GC | 19 (41.3) | 4.00 (0.90–14.00) |  |  |
|  |  | CC | 8 (17.4) | 2.50 (0.90–15.75) |  |  |
|  |  | GC+CC | 27 (58.7) | 3.00 (0.90–14.00) | 1.000 | 0.110 |
|  | rs9676881 | GG | 19 (41.3) | 3.00 (1.30–8.00) | 0.976 | 0.180 |
|  |  | GA | 19 (41.3) | 4.00 (0.90–14.00) |  |  |
|  |  | AA | 8 (17.4) | 2.50 (0.90–15.75) |  |  |
|  |  | GA+AA | 27 (58.7) | 3.00 (0.90–14.00) | 1.000 | 0.110 |
| *HMOX1* | rs2071746 | AA | 16 (34.8) | 3.00 (1.13–10.00) | 0.662 | 0.680 |
|  |  | AT | 21 (45.7) | 1.50 (0.95–13.00) |  |  |
|  |  | TT | 9 (19.6) | 4.00 (1.65–13.00) |  |  |
|  |  | AT+TT | 30 (65.2) | 2.50 (1.00–12.50) | 0.853 | 0.797 |
|  | rs2071747 | GG | 40 (87.0) | 3.00 (1.00–10.00) | 0.313 | 0.425 |
|  |  | GC+CC | 6 (13.0) | 6.50 (1.81–17.00) |  |  |
| *HMOX2* | *rs1051308 | AA | 24 (52.2) | 1.40 (1.00–5.50) | **0.023** | 0.285 |
|  |  | AG | 20 (43.5) | 4.00 (2.00–14.50) |  |  |
|  |  | GG | 2 (4.3) | 23.00 (20.00–26.00) |  |  |
|  |  | AG+GG | 22 (47.8) | 5.00 (2.00–16.00) | **0.031** | 0.225 |
|  | *rs2270363 | GG | 29 (63.0) | 2.00 (1.00–6.00) | **0.048** | 0.475 |
|  |  | GA | 15 (32.6) | 4.00 (1.95–14.50) |  |  |
|  |  | AA | 2 (4.3) | 23.00 (20.00–26.00) |  |  |
|  |  | GA+AA | 17 (37.0) | 10.00 (1.95–16.00) | 0.074 | 0.622 |
| *IL1B* | rs1143623 | CC | 29 (63.0) | 3.00 (0.90–10.00) | 0.608 | 0.234 |
|  |  | CG | 14 (30.4) | 3.00 (2.00–15.00) |  |  |
|  |  | GG | 3 (6.5) | 1.50 (1.38–4.25) |  |  |
|  |  | CG+GG | 17 (37.0) | 3.00 (1.38–14.50) | 0.473 | 0.199 |
|  | *rs16944 | GG | 24 (52.2) | 3.50 (0.93–10.00) | 0.886 | 0.178 |
|  |  | GA | 17 (37.0) | 3.00 (1.50–13.00) |  |  |
|  |  | AA | 5 (10.9) | 1.50 (0.88–16.50) |  |  |
|  |  | GA+AA | 22 (47.8) | 3.00 (1.19–12.50) | 0.800 | 0.074 |
|  | rs1071676 | CC | 26 (56.5) | 3.00 (0.98–10.00) | 0.497 | 0.669 |
|  |  | CG | 15 (32.6) | 4.00 (1.25–16.00) |  |  |
|  |  | GG | 5 (10.9) | 2.00 (0.90–8.00) |  |  |
|  |  | CG+GG | 20 (43.5) | 3.50 (1.26–15.00) | 0.457 | 0.503 |
| *IL6* | *rs1800795 | GG | 8 (17.4) | 7.00 (2.13–15.00) | 0.232 | 0.588 |
|  |  | GC | 25 (84.3) | 1.30 (0.73–11.00) |  |  |
|  |  | CC | 13 (28.3) | 3.00 (1.75–8.50) |  |  |
|  |  | GC+CC | 38 (82.6) | 3.00 (0.98–10.00) | 0.109 | 0.388 |
| *IL6R* | rs2228145 | AA | 16 (34.8) | 2.25 (0.63–11.50) | 0.238 | 0.436 |
|  |  | AC | 22 (47.8) | 5.00 (1.45–14.25) |  |  |
|  |  | CC | 8 (17.4) | 1.65 (1.26–3.75) |  |  |
|  |  | AC+CC | 30 (65.2) | 3.50 (1.30–10.50) | 0.374 | 0.816 |
| *MIR146A* | *rs2910164 | GG | 25 (54.3) | 2.00 (1.00–7.00) | 0.096 | 0.055 |
|  |  | GC | 18 (39.1) | 4.00 (1.30–16.00) |  |  |
|  |  | CC | 3 (6.5) | 8.00 (6.00–24.00) |  |  |
|  |  | GC+CC | 21 (45.7) | 4.00 (1.30–18.00) | 0.059 | **0.020** |
| *TNF* | rs1800629 | GG | 36 (78.3) | 3.00 (1.13–7.50) | **0.031** | 0.530 |
|  |  | GA | 8 (17.4) | 11.00 (5.75–20.00) |  |  |
|  |  | AA | 2 (4.3) | 0.70 (0.50–0.90) |  |  |
|  |  | GA+AA | 10 (21.7) | 10.00 (0.85–20.00) | 0.350 | 0.441 |
| *NLRP3* | rs35829419 | CC | 42 (91.3) | 3.00 (1.00–10.00) | 0.534 | 0.552 |
|  |  | CA+AA | 4 (8.7) | 7.50 (1.50–15.00) |  |  |
| *NOS1* | *rs2293054 | GG | 31 (67.4) | 3.00 (0.90–12.00) | 0.911 | 0.656 |
|  |  | GA | 11 (23.9) | 3.00 (1.30–6.00) |  |  |
|  |  | AA | 4 (8.7) | 2.75 (1.50–16.00) |  |  |
|  |  | GA+AA | 15 (32.6) | 3.00 (1.50–6.00) | 0.869 | 0.598 |
|  | rs2682826 | GG | 25 (54.3) | 4.00 (1.00–14.50) | 0.352 | 0.415 |
|  |  | GA | 15 (32.6) | 2.00 (1.00–4.00) |  |  |
|  |  | AA | 6 (13.0) | 6.00 (1.30–12.50) |  |  |
|  |  | GA+AA | 21 (45.7) | 3.00 (1.13–6.00) | 0.365 | 0.216 |
| *CARD8* | rs2043211 | AA | 21 (45.7) | 1.50 (0.83–10.00) | 0.104 | 0.222 |
|  |  | AT | 21 (45.7) | 4.00 (1.75–15.00) |  |  |
|  |  | TT | 4 (8.7) | 1.80 (0.53–5.25) |  |  |
|  |  | AT+TT | 25 (54.3) | 4.00 (1.38–13.00) | 0.189 | 0.645 |
| *BDNF* | rs6265 | CC | 37 (80.4) | 3.00 (1.30–10.00) | 0.986 | 0.427 |
|  |  | CT | 6 (13.0) | 7.85 (0.50–20.00) |  |  |
|  |  | TT | 3 (6.5) | 1.25 (1.00–20.63) |  |  |
|  |  | CT+TT | 9 (19.6) | 1.25 (0.60–23.00) | 0.892 | 0.266 |
|  | rs28722151 | CC | 15 (32.6) | 3.00 (1.00–4.00) | 0.439 | 0.053 |
|  |  | CG | 20 (43.5) | 3.50 (1.30–11.50) |  |  |
|  |  | GG | 11 (23.9) | 10.00 (1.00–20.00) |  |  |
|  |  | CG+GG | 31 (67.4) | 4.00 (1.25–12.00) | 0.265 | 0.189 |
|  | rs11030101 | AA | 17 (37.0) | 3.00 (1.00–4.00) | 0.677 | 0.099 |
|  |  | AT | 19 (41.3) | 4.00 (1.30–12.00) |  |  |
|  |  | TT | 10 (21.7) | 6.00 (0.94–20.00) |  |  |
|  |  | AT+TT | 29 (63.0) | 4.00 (1.13–13.00) | 0.393 | 0.170 |
| *NOTCH* | rs367398 | GG | 17 (37.0) | 3.00 (0.88–6.00) | 0.478 | 0.818 |
|  |  | GA | 23 (50.0) | 6.00 (1.30–12.00) |  |  |
|  |  | AA | 6 (13.0) | 3.50 (1.10–8.00) |  |  |
|  |  | GA+AA | 29 (63.0) | 4.00 (1.30–12.00) | 0.254 | 0.550 |
| *GSTM1*-present | | | 18 39.1) | 3.00 (1.23–8.50) | 0.919 | 0.354 |
| *GSTM1*-null | | | 28 (60.9) | 3.00 (0.93–13.50) |  |  |
| *GSTT1*-present | | | 38 (82.6) | 3.50 (1.29–12.00) | 0.172 | 0.508 |
| *GSTT1*-null | | | 8 (17.4) | 1.25 (0.63–6.50) |  |  |

*A recessive model was used. Adj: adjusted for *SMN2* copy number. Nominally significant results are printed in bold text.

**Table S5.** Association of all the investigated polymorphisms with RHS score (N = 45).

| **Gene** | **Polymorphism** | **Genotype** | **RHS score, Median (25**–**75%)** | ***p*-value** | ***p*_adj_-value** |
| --- | --- | --- | --- | --- | --- |
| *GSTP1* | rs1695 | AA | 7.50 (2.00–34.50) | 0.246 | 0.729 |
|  |  | AG | 3.00 (0.50–7.50) |  |  |
|  |  | GG | 4.50 (4.00–8.00) |  |  |
|  |  | AG+GG | 4.00 (0.50–8.00) | 0.121 | 0.924 |
|  | rs1138272 | CC | 4.00 (1.25–24.50) | 0.727 | 0.405 |
|  |  | CT+TT | 4.00 (3.00–19.50) |  |  |
| *SOD2* | rs4880 | AA | 4.00 (2.00–9.00) | 0.420 | 0.462 |
|  |  | AG | 8.00 (1.50–33.75) |  |  |
|  |  | GG | 2.50 (1.25–5.75) |  |  |
|  |  | AG+GG | 4.50 (1.75–28.00) | 0.845 | 0.342 |
| *CAT* | rs1001179 | CC | 4.00 (1.00–23.00) | 0.282 | 0.574 |
|  |  | CT | 7.50 (2.00–28.50) |  |  |
|  |  | TT | 2.50 (0.50–3.75) |  |  |
|  |  | CT+TT | 4.50 (2.00–24.50) | 0.774 | 0.883 |
| *GPX1* | rs1050450 | GG | 3.00 (1.00–8.00) | 0.068 | 0.132 |
|  |  | GA | 7.00 (4.00–37.50) |  |  |
|  |  | AA | 0 (0–21.00) |  |  |
|  |  | GA+AA | 6.50 (2.75–42.50) | 0.083 | **0.044** |
| *NFE2L2* | rs6706649 | CC | 4.00 (2.00–16.00) | 0.658 | 0.381 |
|  |  | CT+TT | 6.50 (0.50–39.25) |  |  |
|  | *rs6721961 | GG | 8.00 (2.75–42.50) | **0.025** | 0.172 |
|  |  | GT | 2.00 (0–4.25) |  |  |
|  |  | GT+TT | 2.00 (0–4.00) | **0.007** | 0.064 |
|  | rs35652124 | TT | 4.00 (1.25–7.50) | 0.319 | 0.339 |
|  |  | TC | 4.00 (2.00–45.00) |  |  |
|  |  | CC | 8.00 (3.25–33.00) |  |  |
|  |  | TC+CC | 7.00 (2.00–44.50) | 0.155 | 0.214 |
| *KEAP1* | rs1048290 | GG | 5.50 (3.75–28.00) | 0.450 | 0.906 |
|  |  | GC | 4.00 (1.00–29.00) |  |  |
|  |  | CC | 2.00 (1.25–7.00) |  |  |
|  |  | GC+CC | 3.00 (1.00–15.00) | 0.274 | 0.690 |
|  | rs9676881 | GG | 5.50 (3.75–28.00) | 0.450 | 0.906 |
|  |  | GA | 4.00 (1.00–29.00) |  |  |
|  |  | AA | 2.00 (1.25–7.00) |  |  |
|  |  | GA+AA | 3.00 (1.00–15.00) | 0.876 | 0.690 |
| *HMOX1* | rs2071746 | AA | 4.50 (1.25–16.25) | 0.690 | 0.945 |
|  |  | AT | 4.00 (0.50–21.00) |  |  |
|  |  | TT | 4.50 (2.50–30.50) |  |  |
|  |  | AT+TT | 4.00 (2.00–28.00) | 0.962 | 0.851 |
|  | rs2071747 | GG | 5.00 (2.00–29.00) | 0.286 | **0.006** |
|  |  | GC+CC | 3.00 (1.50–5.25) |  |  |
| *HMOX2* | *rs1051308 | AA | 4.00 (1.50–8.00) | 0.318 | 0.424 |
|  |  | AG | 6.00 (2.00–37.50) |  |  |
|  |  | GG | 28.50 (4.00–53.00) |  |  |
|  |  | AG+GG | 6.00 (2.00–45.00) | 0.189 | 0.215 |
|  | *rs2270363 | GG | 4.00 (1.50–11.00) | 0.323 | 0.597 |
|  |  | GA | 7.00 (2.00–35.50) |  |  |
|  |  | AA | 28.50 (4.00–53.00) |  |  |
|  |  | GA+AA | 7.00 (2.00–46.00) | 0.180 | 0.363 |
| *IL1B* | rs1143623 | CC | 4.00 (2.00–11.00) | 0.535 | 0.358 |
|  |  | CG | 8.50 (1.00–45.00) |  |  |
|  |  | GG | 3.00 (1.50–22.50) |  |  |
|  |  | CG+GG | 8.00 (0.50–43.50) | 0.403 | 0.171 |
|  | *rs16944 | GG | 4.00 (2.00–14.00) | 0.479 | 0.685 |
|  |  | GA | 8.00 (1.50–29.00) |  |  |
|  |  | AA | 4.00 (1.50–47.50) |  |  |
|  |  | GA+AA | 7.00 (1.75–33.75) | 0.226 | 0.386 |
|  | rs1071676 | CC | 4.00 (2.50–21.00) | 0.545 | 0.371 |
|  |  | CG | 2.00 (0–29.00) |  |  |
|  |  | GG | 5.00 (2.00–24.00) |  |  |
|  |  | CG+GG | 3.00 (0–26.00) | 0.383 | 0.272 |
| *IL6* | *rs1800795 | GG | 4.00 (2.00–14.75) | 0.987 | 0.478 |
|  |  | GC | 4.00 (0.50–24.50) |  |  |
|  |  | CC | 5.00 (1.50–36.50) |  |  |
|  |  | GC+CC | 4.00 (1.50–28.00) | 1.000 | 0.281 |
| *IL6R* | rs2228145 | AA | 4.00 (2.00–14.00) | 0.749 | 0.304 |
|  |  | AC | 5.00 (1.00–42.75) |  |  |
|  |  | CC | 4.50 (2.50–8.00) |  |  |
|  |  | AC+CC | 4.50 (1.75–29.50) | 0.483 | 0.188 |
| *MIR146A* | *rs2910164 | GG | 4.00 (0.50–8.50) | 0.150 | 0.107 |
|  |  | GC | 4.00 (2.00–48.00) |  |  |
|  |  | CC | 29.00 (17.50–37.00) |  |  |
|  |  | GC+CC | 6.00 (2.00–46.50) | 0.117 | **0.034** |
| *TNF* | rs1800629 | GG | 4.00 (1.50–12.00) | 0.300 | 0.350 |
|  |  | GA | 9.00 (4.00–35.00) |  |  |
|  |  | AA | 22.00 (0–44.00) |  |  |
|  |  | GA+AA | 9.00 (3.75–46.25) | 0.179 | 0.394 |
| *NLRP3* | rs35829419 | CC | 4.00 (2.00–21.00) | 1.000 | 0.902 |
|  |  | CA+AA | 9.50 (0.50–40.25) |  |  |
| *NOS1* | *rs2293054 | GG | 4.00 (0.75–19.50) | 0.815 | 0.469 |
|  |  | GA | 7.00 (2.00–31.00) |  |  |
|  |  | AA | 4.00 (2.25–44.75) |  |  |
|  |  | GA+AA | 5.00 (2.00–31.00) | 0.529 | 0.249 |
|  | rs2682826 | GG | 4.00 (0.50–28.00) | 0.449 | 0.518 |
|  |  | GA | 3.50 (1.75–9.50) |  |  |
|  |  | AA | 6.00 (3.50–48.25) |  |  |
|  |  | GA+AA | 4.00 (2.00–12.50) | 0.890 | 0.700 |
| *CARD8* | rs2043211 | AA | 4.00 (2.25–7.75) | 0.690 | 0.722 |
|  |  | AT | 8.00 (1.50–36.50) |  |  |
|  |  | TT | 2.50 (0.50–39.00) |  |  |
|  |  | AT+TT | 6.00 (1.50–36.50) | 0.654 | 0.420 |
| *BDNF* | rs6265 | CC | 4.00 (2.00–15.00) | 0.919 | 0.595 |
|  |  | CT | 4.00 (4.00–27.00) |  |  |
|  |  | TT | 14.50 (0–29.00) |  |  |
|  |  | CT+TT | 4.00 (1.00–28.50) | 0.919 | 0.439 |
|  | rs28722151 | CC | 2.00 (1.00–8.00) | 0.421 | 0.708 |
|  |  | CG | 4.50 (2.25–24.50) |  |  |
|  |  | GG | 5.50 (1.50–36.75) |  |  |
|  |  | CG+GG | 4.50 (2.00–29.50) | 0.195 | 0.416 |
|  | rs11030101 | AA | 3.00 (1.50–8.50) | 0.585 | 0.437 |
|  |  | AT | 4.00 (2.00–42.00) |  |  |
|  |  | TT | 4.00 (1.00–30.00) |  |  |
|  |  | AT+TT | 4.00 (2.00–30.50) | 0.323 | 0.323 |
| *NOTCH* | rs367398 | GG | 2.00 (0–7.50) | **0.028** | **0.037** |
|  |  | GA | 14.00 (3.00–44.00) |  |  |
|  |  | AA | 4.00 (1.50–5.75) |  |  |
|  |  | GA+AA | 5.00 (2.50–36.50) | **0.025** | **0.050** |
| *GSTM1*–present | | | 5.50 (3.00–34.50) | 0.204 | 0.129 |
| *GSTM1*–null | | | 4.00 (1.00–15.00) |  |  |
| *GSTT1*–present | | | 5.00 (2.00–22.00) | 0.405 | 0.979 |
| *GSTT1*–null | | | 3.00 (0.50–22.75) |  |  |

*A recessive model was used. Adj: adjusted for age, *SMN2* copy number, and disease duration. RHS: Revised Hammersmith Scale. Nominally significant results are printed in bold text.

**Table S6.** Association of all the investigated polymorphisms with RULM score (N = 44).

| **Gene** | **Polymorphism** | **Genotype** | **RULM score, Median (25**–**75%)** | ***p*-value** | ***p*_adj_-value** |
| --- | --- | --- | --- | --- | --- |
| *GSTP1* | rs1695 | AA | 22.00 (16.00–33.00) | 0.082 | 0.106 |
|  |  | AG | 14.00 (7.00–21.00) |  |  |
|  |  | GG | 24.50 (9.75–27.50) |  |  |
|  |  | AG+GG | 15.00 (7.50–24.50) | 0.052 | 0.356 |
|  | rs1138272 | CC | 18.00 (11.00–27.00) | 0.932 | 0.598 |
|  |  | CT+TT | 19.00 (10.50–27.00) |  |  |
| *SOD2* | rs4880 | AA | 13.00 (9.00–24.00) | 0.768 | 0.682 |
|  |  | AG | 19.00 (8.00–32.50) |  |  |
|  |  | GG | 19.50 (15.25–23.75) |  |  |
|  |  | AG+GG | 19.00 (14.00–30.50) | 0.504 | 0.682 |
| *CAT* | rs1001179 | CC | 18.00 (8.50–27.00) | 0.067 | 0.141 |
|  |  | CT | 22.00 (15.00–32.00) |  |  |
|  |  | TT | 10.00 (2.25–16.25) |  |  |
|  |  | CT+TT | 19.00 (14.00–27.00) | 0.548 | 0.442 |
| *GPX1* | rs1050450 | GG | 18.00 (9.00–24.50) | 0.089 | 0.100 |
|  |  | GA | 22.00 (17.00–33.00) |  |  |
|  |  | AA | 7.50 (2.00–13.00) |  |  |
|  |  | GA+AA | 20.00 (13.50–33.00) | 0.290 | 0.154 |
| *NFE2L2* | rs6706649 | CC | 18.00 (11.00–24.50) | 0.669 | 0.593 |
|  |  | CT+TT | 21.00 (11.00–29.00) |  |  |
|  | *rs6721961 | GG | 21.00 (13.50–33.50) | 0.143 | 0.532 |
|  |  | GT | 15.50 (9.00–22.25) |  |  |
|  |  | GT+TT | 15.00 (9.00–22.00) | 0.053 | 0.259 |
|  | rs35652124 | TT | 19.00 (13.00–24.00) | 0.736 | 0.588 |
|  |  | TC | 21.00 (9.00–37.00) |  |  |
|  |  | CC | 18.00 (13.25–26.50) |  |  |
|  |  | TC+CC | 18.00 (9.00–35.00) | 0.484 | 0.906 |
| *KEAP1* | rs1048290 | GG | 23.00 (14.00–30.50) | 0.525 | 0.503 |
|  |  | GC | 18.00 (7.00–24.00) |  |  |
|  |  | CC | 15.50 (9.50–25.75) |  |  |
|  |  | GC+CC | 18.00 (9.00–24.00) | 0.257 | 0.288 |
|  | rs9676881 | GG | 23.00 (14.00–30.50) | 0.525 | 0.503 |
|  |  | GA | 18.00 (7.00–24.00) |  |  |
|  |  | AA | 15.50 (9.50–25.75) |  |  |
|  |  | GA+AA | 18.00 (9.00–24.00) | 0.257 | 0.288 |
| *HMOX1* | rs2071746 | AA | 20.00 (10.00–32.75) | 0.633 | 0.654 |
|  |  | AT | 18.00 (9.50–23.75) |  |  |
|  |  | TT | 22.50 (15.25–27.75) |  |  |
|  |  | AT+TT | 18.50 (11.50–24.00) | 0.625 | 0.560 |
|  | rs2071747 | GG | 18.50 (12.50–29.75) | 0.451 | 0.063 |
|  |  | GC+CC | 18.00 (8.25–22.50) |  |  |
| *HMOX2* | *rs1051308 | AA | 16.50 (9.00–25.00) | 0.247 | 0.715 |
|  |  | AG | 21.00 (14.00–31.50) |  |  |
|  |  | GG | 29.00 (21.00–37.00) |  |  |
|  |  | AG+GG | 21.50 (14.50–34.50) | 0.173 | 0.415 |
|  | *rs2270363 | GG | 18.00 (9.00–25.00) | 0.302 | 0.881 |
|  |  | GA | 20.00 (15.50–30.00) |  |  |
|  |  | AA | 29.00 (21.00–37.00) |  |  |
|  |  | GA+AA | 21.00 (15.50–36.50) | 0.218 | 0.621 |
| *IL1B* | rs1143623 | CC | 18.00 (10.00–24.50) | 0.137 | 0.230 |
|  |  | CG | 23.00 (14.00–36.00) |  |  |
|  |  | GG | 10.00 (6.00–14.00) |  |  |
|  |  | CG+GG | 20.50 (13.25–34.25) | 0.366 | 0.784 |
|  | *rs16944 | GG | 18.00 (8.00–25.00) | 0.449 | 0.597 |
|  |  | GA | 22.00 (13.50–28.00) |  |  |
|  |  | AA | 18.00 (8.00–33.25) |  |  |
|  |  | GA+AA | 22.00 (13.50–28.00) | 0.244 | 0.542 |
|  | rs1071676 | CC | 18.50 (10.00–24.50) | 0.681 | 0.308 |
|  |  | CG | 16.00 (11.00–32.00) |  |  |
|  |  | GG | 25.00 (12.00–31.00) |  |  |
|  |  | CG+GG | 21.00 (11.50–31.25) | 0.662 | 0.745 |
| *IL6* | *rs1800795 | GG | 19.00 (12.00–29.75) | 0.937 | 0.693 |
|  |  | GC | 18.50 (9.00–26.50) |  |  |
|  |  | CC | 20.00 (13.25–28.00) |  |  |
|  |  | GC+CC | 18.50 (10.00–26.50) | 0.917 | 0.834 |
| *IL6R* | rs2228145 | AA | 18.00 (11.00–32.00) | 0.980 | 0.849 |
|  |  | AC | 20.00 (11.00–29.00) |  |  |
|  |  | CC | 21.50 (10.00–26.50) |  |  |
|  |  | AC+CC | 20.00 (11.00–26.00) | 0.852 | 0.621 |
| *MIR146A* | *rs2910164 | GG | 18.00 (9.00–23.00) | 0.070 | 0.062 |
|  |  | GC | 21.50 (14.00–36.00) |  |  |
|  |  | CC | 24.00 (23.50–30.50) |  |  |
|  |  | GC+CC | 23.00 (14.50–36.50) | **0.036** | **0.018** |
| *TNF* | rs1800629 | GG | 18.00 (11.00–24.00) | 0.460 | 0.360 |
|  |  | GA | 26.50 (13.50–35.00) |  |  |
|  |  | AA | 23.00 (9.00–37.00) |  |  |
|  |  | GA+AA | 26.50 (12.00–37.00) | 0.227 | 0.216 |
| *NLRP3* | rs35829419 | CC | 18.50 (11.50–25.00) | 0.738 | 0.991 |
|  |  | CA+AA | 24.00 (5.25–36.00) |  |  |
| *NOS1* | *rs2293054 | GG | 18.00 (10.00–24.50) | 0.706 | 0.407 |
|  |  | GA | 19.00 (9.00–29.00) |  |  |
|  |  | AA | 21.00 (15.00–33.75) |  |  |
|  |  | GA+AA | 19.00 (14.00–29.00) | 0.472 | 0.229 |
|  | rs2682826 | GG | 19.00 (9.00–24.75) | 0.514 | 0.272 |
|  |  | GA | 18.50 (12.00–27.50) |  |  |
|  |  | AA | 21.00 (16.75–37.00) |  |  |
|  |  | GA+AA | 18.50 (13.25–28.50) | 0.450 | 0.133 |
| *CARD8* | rs2043211 | AA | 18.00 (13.00–24.75) | 0.723 | 0.793 |
|  |  | AT | 22.50 (9.50–32.00) |  |  |
|  |  | TT | 16.50 (3.75–30.00) |  |  |
|  |  | AT+TT | 21.00 (9.50–32.00) | 0.688 | 0.914 |
| *BDNF* | rs6265 | CC | 18.50 (12.00–28.00) | 0.818 | 0.206 |
|  |  | CT | 19.50 (13.00–22.00) |  |  |
|  |  | TT | 15.00 (6.00–24.00) |  |  |
|  |  | CT+TT | 19.50 (7.75–23.50) | 0.622 | 0.122 |
|  | rs28722151 | CC | 16.00 (7.00–24.00) | 0.513 | 0.635 |
|  |  | CG | 21.00 (15.00–32.00) |  |  |
|  |  | GG | 16.00 (10.50–30.75) |  |  |
|  |  | CG+GG | 20.00 (13.00–30.50) | 0.327 | 0.571 |
|  | rs11030101 | AA | 19.00 (8.00–26.00) | 0.564 | 0.567 |
|  |  | AT | 20.50 (14.50–33.25) |  |  |
|  |  | TT | 14.00 (10.00–26.50) |  |  |
|  |  | AT+TT | 18.00 (13.00–29.00) | 0.699 | 0.912 |
| *NOTCH* | rs367398 | GG | 14.50 (9.00–18.75) | **0.047** | **0.031** |
|  |  | GA | 23.00 (14.75–34.50) |  |  |
|  |  | AA | 22.50 (5.25–25.50) |  |  |
|  |  | GA+AA | 23.00 (14.25–32.75) | **0.022** | **0.011** |
| *GSTM1*-present | | | 23.50 (13.75–33.25) | 0.107 | **0.030** |
| *GSTM1*-null | | | 18.00 (8.75–24.00) |  |  |
| *GSTT1*-present | | | 19.50 (9.50–28.50) | 0.643 | 0.941 |
| *GSTT1*-null | | | 16.00 (13.25–23.50) |  |  |

*A recessive model was used. Adj: adjusted for age, *SMN2* copy number, and disease duration. RULM: Revised Upper Limb Module. Nominally significant results are printed in bold text.

**Table S7.** Association of all the investigated polymorphisms with VC (%) (N = 45).

| **Gene** | **Polymorphism** | **Genotype** | **VC (%), Median (25**–**75%)** | ***p*-value** | ***p*_adj_-value** |
| --- | --- | --- | --- | --- | --- |
| *GSTP1* | rs1695 | AA | 84.50 (41.50–95.25) | 0.216 | 0.633 |
|  |  | AG | 61.00 (32.00–85.00) |  |  |
|  |  | GG | 57.00 (21.75–80.25) |  |  |
|  |  | AG+GG | 61.00 (27.50–80.50) | 0.087 | 0.938 |
|  | rs1138272 | CC | 66.00 (38.25–90.50) | 0.459 | 0.247 |
|  |  | CT+TT | 64.00 (30.50–76.50) |  |  |
| *SOD2* | rs4880 | AA | 64.00 (39.00–87.00) | 0.306 | 0.614 |
|  |  | AG | 80.50 (38.00–96.75) |  |  |
|  |  | GG | 56.00 (35.25–72.25) |  |  |
|  |  | AG+GG | 64.00 (38.00–89.50) | 0.927 | 0.815 |
| *CAT* | rs1001179 | CC | 61.00 (35.00–87.00) | 0.189 | 0.084 |
|  |  | CT | 73.00 (45.00–98.75) |  |  |
|  |  | TT | 39.50 (21.75–75.25) |  |  |
|  |  | CT+TT | 71.50 (39.75–95.75) | 0.333 | 0.205 |
| *GPX1* | rs1050450 | GG | 58.00 (38.00–76.00) | 0.132 | 0.095 |
|  |  | GA | 85.00 (56.50–92.00) |  |  |
|  |  | AA | 17.00 (16.00–61.00) |  |  |
|  |  | GA+AA | 83.00 (37.50–93.75) | 0.140 | 0.116 |
| *NFE2L2* | rs6706649 | CC | 67.00 (38.50–90.00) | 0.409 | 0.192 |
|  |  | CT+TT | 45.50 (25.25–81.75) |  |  |
|  | *rs6721961 | GG | 77.00 (41.00–93.75) | 0.189 | 0.646 |
|  |  | GT | 56.50 (36.50–74.50) |  |  |
|  |  | GT+TT | 54.00 (32.00–71.00) | 0.115 | 0.427 |
|  | rs35652124 | TT | 41.00 (32.25–78.00) | 0.089 | 0.290 |
|  |  | TC | 74.00 (45.00–96.00) |  |  |
|  |  | CC | 88.00 (62.50–97.25) |  |  |
|  |  | TC+CC | 81.00 (52.00–94.50) | 0.059 | 0.691 |
| *KEAP1* | rs1048290 | GG | 68.00 (38.00–94.50) | 0.928 | 0.715 |
|  |  | GC | 61.00 (33.00–89.00) |  |  |
|  |  | CC | 60.50 (40.00–77.75) |  |  |
|  |  | GC+CC | 61.00 (38.00–88.00) | 0.880 | 0.605 |
|  | rs9676881 | GG | 68.00 (38.00–94.50) | 0.928 | 0.715 |
|  |  | GA | 61.00 (33.00–89.00) |  |  |
|  |  | AA | 60.50 (40.00–77.75) |  |  |
|  |  | GA+AA | 61.00 (38.00–88.00) | 0.880 | 0.605 |
| *HMOX1* | rs2071746 | AA | 66.00 (34.25–94.25) | 0.805 | 0.774 |
|  |  | AT | 67.00 (35.00–87.50) |  |  |
|  |  | TT | 61.50 (48.50–90.00) |  |  |
|  |  | AT+TT | 64.00 (40.00–87.50) | 0.758 | 0.939 |
|  | rs2071747 | GG | 67.00 (38.00–91.00) | 0.568 | **0.028** |
|  |  | GC+CC | 56.50 (36.25–85.50) |  |  |
| *HMOX2* | *rs1051308 | AA | 61.00 (35.50–82.50) | 0.611 | 0.243 |
|  |  | AG | 76.00 (44.00–90.00) |  |  |
|  |  | GG | 70.50 (45.00–96.00) |  |  |
|  |  | AG+GG | 76.00 (44.25–91.50) | 0.334 | 0.524 |
|  | *rs2270363 | GG | 62.50 (38.00–83.00) | 0.739 | 0.233 |
|  |  | GA | 82.00 (40.50–90.00) |  |  |
|  |  | AA | 70.50 (45.00–96.00) |  |  |
|  |  | GA+AA | 82.00 (40.50–93.50) | 0.461 | 0.406 |
| *IL1B* | rs1143623 | CC | 62.50 (40.50–88.00) | 0.808 | 0.697 |
|  |  | CG | 72.50 (38.00–88.00) |  |  |
|  |  | GG | 38.00 (24.00–71.50) |  |  |
|  |  | CG+GG | 71.00 (38.00–92.00) | 0.935 | 0.695 |
|  | *rs16944 | GG | 61.00 (42.00–89.00) | 0.946 | 0.631 |
|  |  | GA | 74.00 (38.00–87.50) |  |  |
|  |  | AA | 39.00 (24.00–100.50) |  |  |
|  |  | GA+AA | 72.50 (38.00–88.75) | 0.964 | 0.899 |
|  | rs1071676 | CC | 64.00 (38.50–90.00) | 0.895 | 0.079 |
|  |  | CG | 59.00 (32.00–87.00) |  |  |
|  |  | GG | 72.00 (25.00–90.00) |  |  |
|  |  | CG+GG | 63.00 (33.50–85.75) | 0.640 | 0.105 |
| *IL6* | *rs1800795 | GG | 74.50 (58.75–90.00) | 0.427 | 0.731 |
|  |  | GC | 52.00 (32.25–91.00) |  |  |
|  |  | CC | 71.00 (39.00–88.50) |  |  |
|  |  | GC+CC | 59.00 (35.50–88.50) | 0.234 | 0.541 |
| *IL6R* | rs2228145 | AA | 46.00 (33.00–96.00) | 0.473 | 0.455 |
|  |  | AC | 78.00 (38.75–91.50) |  |  |
|  |  | CC | 61.50 (39.00–78.00) |  |  |
|  |  | AC+CC | 71.50 (38.75–87.25) | 0.605 | 0.209 |
| *MIR146A* | *rs2910164 | GG | 50.50 (35.00–86.00) | 0.143 | 0.479 |
|  |  | GC | 62.50 (46.00–93.00) |  |  |
|  |  | CC | 87.00 (80.50–111.00) |  |  |
|  |  | GC+CC | 74.00 (50.00–94.50) | 0.122 | 0.323 |
| *TNF* | rs1800629 | GG | 61.00 (38.50–84.50) | 0.400 | 0.619 |
|  |  | GA | 90.50 (41.50–99.50) |  |  |
|  |  | AA | 68.00 (38.00–98.00) |  |  |
|  |  | GA+AA | 90.50 (38.00–99.25) | 0.198 | 0.472 |
| *NLRP3* | rs35829419 | CC | 64.00 (38.00–88.50) | 0.923 | 0.405 |
|  |  | CA+AA | 70.50 (27.50–94.75) |  |  |
| *NOS1* | *rs2293054 | GG | 58.50 (32.75–92.25) | 0.955 | 0.309 |
|  |  | GA | 72.00 (59.00–82.00) |  |  |
|  |  | AA | 54.50 (39.00–91.75) |  |  |
|  |  | GA+AA | 71.00 (42.00–82.00) | 0.800 | 0.158 |
|  | rs2682826 | GG | 64.00 (39.00–94.50) | 0.492 | 0.413 |
|  |  | GA | 60.00 (36.75–80.25) |  |  |
|  |  | AA | 70.50 (37.25–91.75) |  |  |
|  |  | GA+AA | 64.00 (38.00–80.75) | 0.392 | 0.461 |
| *CARD8* | rs2043211 | AA | 51.50 (38.00–88.00) | 0.355 | 0.600 |
|  |  | AT | 80.00 (50.00–89.50) |  |  |
|  |  | TT | 46.00 (14.25–84.50) |  |  |
|  |  | AT+TT | 74.00 (39.50–89.50) | 0.458 | 0.850 |
| *BDNF* | rs6265 | CC | 67.00 (40.00–87.00) | 0.800 | 0.099 |
|  |  | CT | 42.00 (23.00–96.00) |  |  |
|  |  | TT | 72.50 (10.00–135.00) |  |  |
|  |  | CT+TT | 42.00 (13.25–106.50) | 0.590 | **0.032** |
|  | rs28722151 | CC | 59.00 (38.00–82.00) | 0.653 | 0.487 |
|  |  | CG | 65.50 (39.25–95.25) |  |  |
|  |  | GG | 83.00 (22.50–91.75) |  |  |
|  |  | CG+GG | 69.50 (38.75–93.75) | 0.360 | 0.306 |
|  | rs11030101 | AA | 59.00 (38.00–84.50) | 0.797 | 0.459 |
|  |  | AT | 67.00 (39.00–96.00) |  |  |
|  |  | TT | 81.00 (22.00–88.50) |  |  |
|  |  | AT+TT | 69.50 (38.25–92.50) | 0.543 | 0.516 |
| *NOTCH* | rs367398 | GG | 52.50 (24.00–73.25) | **0.049** | 0.135 |
|  |  | GA | 85.00 (40.00–98.00) |  |  |
|  |  | AA | 53.00 (33.50–74.00) |  |  |
|  |  | GA+AA | 80.00 (41.00–94.50) | 0.064 | 0.084 |
| *GSTM1*-present | | | 73.00 (36.75–85.50) | 0.754 | 0.381 |
| *GSTM1*-null | | | 61.00 (39.00–91.00) |  |  |
| *GSTT1*-present | | | 71.00 (41.00–90.00) | 0.121 | 0.368 |
| *GSTT1*-null | | | 35.50 (25.25–69.00) |  |  |

*A recessive model was used. Adj: adjusted for age, *SMN2* copy number, and disease duration. VC (%): vital capacity percent predicted. Nominally significant results are printed in bold text.

**Table S8.** Association of all the investigated polymorphisms with PEF (%) (N = 45).

| **Gene** | **Polymorphism** | **Genotype** | **PEF (%), Median (25**–**75%)** | ***p*-*value*** | ***p*_adj_-value** |
| --- | --- | --- | --- | --- | --- |
| *GSTP1* | rs1695 | AA | 69.00 (50.25–88.25) | 0.470 | 0.415 |
|  |  | AG | 62.00 (40.00–82.00) |  |  |
|  |  | GG | 60.50 (41.75–82.75) |  |  |
|  |  | AG+GG | 62.00 (41.00–81.00) | 0.221 | 0.985 |
|  | rs1138272 | CC | 61.00 (47.00–86.00) | 0.791 | 0.266 |
|  |  | CT+TT | 64.00 (46.00–80.50) |  |  |
| *SOD2* | rs4880 | AA | 58.00 (50.00–89.00) | 0.398 | 0.777 |
|  |  | AG | 73.50 (44.50–94.00) |  |  |
|  |  | GG | 51.50 (43.25–76.00) |  |  |
|  |  | AG+GG | 63.00 (45.00–84.50) | 0.825 | 0.972 |
| *CAT* | rs1001179 | CC | 60.00 (46.50–83.00) | 0.163 | 0.206 |
|  |  | CT | 75.00 (51.00–91.25) |  |  |
|  |  | TT | 45.50 (22.75–63.75) |  |  |
|  |  | CT+TT | 66.00 (48.00–86.00) | 0.625 | 0.603 |
| *GPX1* | rs1050450 | GG | 52.00 (46.50–76.00) | 0.183 | 0.112 |
|  |  | GA | 80.00 (52.50–85.00) |  |  |
|  |  | AA | 42.00 (30.50–81.00) |  |  |
|  |  | GA+AA | 78.50 (50.75–87.75) | 0.122 | **0.045** |
| *NFE2L2* | rs6706649 | CC | 64.00 (50.50–85.00) | 0.502 | 0.763 |
|  |  | CT+TT | 50.50 (40.50–90.25) |  |  |
|  | *rs6721961 | GG | 78.50 (50.00–90.00) | 0.132 | 0.598 |
|  |  | GT | 51.50 (45.00–65.00) |  |  |
|  |  | GT+TT | 51.00 (46.00–64.00) | **0.047** | 0.319 |
|  | rs35652124 | TT | 51.50 (40.50–68.50) | **0.036** | 0.123 |
|  |  | TC | 68.00 (47.00–86.00) |  |  |
|  |  | CC | 86.50 (77.50–101.75) |  |  |
|  |  | TC+CC | 80.00 (49.00–87.50) | 0.093 | 0.747 |
| *KEAP1* | rs1048290 | GG | 73.50 (45.25–86.00) | 0.934 | 0.537 |
|  |  | GC | 62.00 (42.00–84.00) |  |  |
|  |  | CC | 51.50 (50.25–85.75) |  |  |
|  |  | GC+CC | 58.00 (47.00–84.00) | 0.711 | 0.432 |
|  | rs9676881 | GG | 73.50 (45.25–86.00) | 0.934 | 0.537 |
|  |  | GA | 62.00 (42.00–84.00) |  |  |
|  |  | AA | 51.50 (50.25–85.75) |  |  |
|  |  | GA+AA | 58.00 (47.00–84.00) | 0.711 | 0.432 |
| *HMOX1* | rs2071746 | AA | 61.00 (44.00–86.00) | 0.806 | 0.826 |
|  |  | AT | 64.00 (43.00–83.00) |  |  |
|  |  | TT | 64.50 (48.00–92.25) |  |  |
|  |  | AT+TT | 64.00 (47.00–84.00) | 0.794 | 0.656 |
|  | rs2071747 | GG | 64.00 (50.00–86.00) | 0.257 | **0.009** |
|  |  | GC+CC | 49.00 (38.25–73.25) |  |  |
| *HMOX2* | *rs1051308 | AA | 64.00 (44.00–82.00) | 0.803 | 0.699 |
|  |  | AG | 55.50 (51.00–86.50) |  |  |
|  |  | GG | 84.00 (47.00–121.00) |  |  |
|  |  | AG+GG | 55.50 (50.00–90.00) | 0.658 | 0.432 |
|  | *rs2270363 | GG | 63.00 (48.00–82.00) | 0.829 | 0.691 |
|  |  | GA | 58.00 (49.00–86.50) |  |  |
|  |  | AA | 84.00 (47.00–121.00) |  |  |
|  |  | GA+AA | 58.00 (47.00–91.00) | 0.725 | 0.411 |
| *IL1B* | rs1143623 | CC | 63.00 (48.50–83.00) | 0.979 | 0.979 |
|  |  | CG | 64.50 (46.00–89.00) |  |  |
|  |  | GG | 60.00 (36.00–90.00) |  |  |
|  |  | CG+GG | 60.00 (43.00–91.00) | 0.888 | 0.991 |
|  | *rs16944 | GG | 64.00 (47.00–84.00) | 0.799 | 0.894 |
|  |  | GA | 53.00 (48.00–86.50) |  |  |
|  |  | AA | 60.00 (35.00–120.50) |  |  |
|  |  | GA+AA | 59.00 (49.00–90.00) | 0.699 | 0.709 |
|  | rs1071676 | CC | 62.00 (50.00–88.50) | 0.713 | 0.065 |
|  |  | CG | 52.00 (37.00–86.00) |  |  |
|  |  | GG | 70.00 (34.50–81.50) |  |  |
|  |  | CG+GG | 58.00 (39.25–85.00) | 0.417 | 0.050 |
| *IL6* | *rs1800795 | GG | 64.00 (55.25–81.50) | 0.759 | 0.928 |
|  |  | GC | 54.50 (40.50–91.25) |  |  |
|  |  | CC | 60.00 (50.50–86.50) |  |  |
|  |  | GC+CC | 58.00 (44.00–87.50) | 0.550 | 0.806 |
| *IL6R* | rs2228145 | AA | 50.00 (46.00–86.00) | 0.461 | 0.553 |
|  |  | AC | 66.00 (51.75–90.00) |  |  |
|  |  | CC | 61.00 (42.75–82.25) |  |  |
|  |  | AC+CC | 66.00 (51.00–85.25) | 0.300 | 0.274 |
| *MIR146A* | *rs2910164 | GG | 52.50 (44.00–73.50) | 0.129 | 0.411 |
|  |  | GC | 73.00 (51.00–93.00) |  |  |
|  |  | CC | 80.00 (80.00–87.50) |  |  |
|  |  | GC+CC | 80.00 (51.00–93.00) | 0.069 | 0.180 |
| *TNF* | rs1800629 | GG | 58.00 (44.50–82.00) | 0.419 | 0.532 |
|  |  | GA | 77.00 (55.00–89.50) |  |  |
|  |  | AA | 69.50 (46.00–93.00) |  |  |
|  |  | GA+AA | 77.00 (49.25–93.00) | 0.218 | 0.322 |
| *NLRP3* | rs35829419 | CC | 62.00 (48.50–85.00) | 0.863 | 0.489 |
|  |  | CA+AA | 64.50 (43.25–85.00) |  |  |
| *NOS1* | *rs2293054 | GG | 55.50 (45.00–86.00) | 0.801 | 0.489 |
|  |  | GA | 70.00 (52.00–82.00) |  |  |
|  |  | AA | 62.00 (53.25–85.75) |  |  |
|  |  | GA+AA | 64.00 (52.00–82.00) | 0.508 | 0.268 |
|  | rs2682826 | GG | 64.00 (46.50–91.00) | 0.661 | 0.648 |
|  |  | GA | 59.00 (40.00–78.25) |  |  |
|  |  | AA | 72.00 (50.75–84.75) |  |  |
|  |  | GA+AA | 61.00 (50.25–81.50) | 0.656 | 0.861 |
| *CARD8* | rs2043211 | AA | 59.00 (47.75–83.50) | 0.416 | 0.458 |
|  |  | AT | 77.00 (51.00–87.50) |  |  |
|  |  | TT | 43.50 (23.50–84.50) |  |  |
|  |  | AT+TT | 64.00 (44.50–87.50) | 0.766 | 0.456 |
| *BDNF* | rs6265 | CC | 64.00 (50.00–84.00) | 0.961 | 0.168 |
|  |  | CT | 54.00 (47.00–116.00) |  |  |
|  |  | TT | 53.50 (12.00–95.00) |  |  |
|  |  | CT+TT | 54.00 (25.25–110.75) | 0.873 | 0.262 |
|  | rs28722151 | CC | 52.00 (42.00–82.00) | 0.706 | 0.529 |
|  |  | CG | 64.00 (47.75–86.00) |  |  |
|  |  | GG | 72.50 (46.75–86.25) |  |  |
|  |  | CG+GG | 66.00 (49.25–86.00) | 0.420 | 0.555 |
|  | rs11030101 | AA | 52.00 (44.50–84.00) | 0.625 | 0.377 |
|  |  | AT | 64.00 (47.00–93.00) |  |  |
|  |  | TT | 68.00 (43.50–83.00) |  |  |
|  |  | AT+TT | 66.00 (47.75–85.50) | 0.475 | 0.625 |
| *NOTCH* | rs367398 | GG | 49.00 (40.00–80.00) | 0.067 | 0.212 |
|  |  | GA | 77.00 (53.00–93.00) |  |  |
|  |  | AA | 56.50 (39.75–77.50) |  |  |
|  |  | GA+AA | 68.00 (51.00–89.50) | **0.044** | 0.094 |
| *GSTM1*-present | | | 69.00 (48.00–83.00) | 0.668 | 0.267 |
| *GSTM1*-null | | | 53.00 (47.00–86.00) |  |  |
| *GSTT1*-present | | | 64.00 (48.50–86.00) | 0.190 | 0.559 |
| *GSTT1*-null | | | 50.50 (37.75–75.00) |  |  |

*A recessive model was used. Adj: adjusted for age, *SMN2* copy number, and disease duration. PEF (%): peak expiratory flow percent predicted. Nominally significant results are printed in bold text.

**Table S9.** DNA quality metrics for each experimental group.

| **Experimental Group** | **Sample ID** | **DNA Concentration (ng/µL)** | **260/280 Ratio** |
| --- | --- | --- | --- |
| SMA patient | Sample 1 | 27 | 2.158 |
| SMA patient | Sample 2 | 20.9 | 2.217 |
| SMA patient | Sample 3 | 6.7 | 2.107 |
| SMA patient | Sample 4 | 27.5 | 2.114 |
| SMA patient | Sample 5 | 163.3 | 1.881 |
| SMA patient | Sample 6 | 14.8 | 2.172 |
| SMA patient | Sample 7 | 414.4 | 1.851 |
| SMA patient | Sample 8 | 29.7 | 2.095 |
| SMA patient | Sample 9 | 41.1 | 2.096 |
| SMA patient | Sample 10 | 365.2 | 1.885 |
| SMA patient | Sample 11 | 18.1 | 2.099 |
| SMA patient | Sample 12 | 20.5 | 2.155 |
| SMA patient | Sample 13 | 14.2 | 2.284 |
| SMA patient | Sample 14 | 60.3 | 2.024 |
| SMA patient | Sample 15 | 36.9 | 2.121 |
| SMA patient | Sample 16 | 10.9 | 2.475 |
| SMA patient | Sample 17 | 16.5 | 2.193 |
| SMA patient | Sample 18 | 14.2 | 2.121 |
| SMA patient | Sample 19 | 31.1 | 2.058 |
| SMA patient | Sample 20 | 438.7 | 1.894 |
| SMA patient | Sample 21 | 20.6 | 2.142 |
| SMA patient | Sample 22 | 21 | 2.001 |
| SMA patient | Sample 23 | 54.7 | 2.047 |
| SMA patient | Sample 24 | 14.7 | 2.187 |
| SMA patient | Sample 25 | 37.5 | 1.875 |
| SMA patient | Sample 26 | 32 | 1.937 |
| SMA patient | Sample 27 | 28.6 | 2.029 |
| SMA patient | Sample 28 | 44.7 | 2.062 |
| SMA patient | Sample 29 | 527.8 | 1.796 |
| SMA patient | Sample 30 | 25.0 | 1.998 |
| SMA patient | Sample 31 | 49.2 | 1.991 |
| SMA patient | Sample 32 | 37.6 | 2.019 |
| SMA patient | Sample 33 | 12.2 | 2.339 |
| SMA patient | Sample 34 | 26.1 | 1.919 |
| SMA patient | Sample 35 | 274.6 | 1.899 |
| SMA patient | Sample 36 | 274.5 | 1.893 |
| SMA patient | Sample 37 | 42.1 | 1.997 |
| SMA patient | Sample 38 | 14.4 | 1.820 |
| SMA patient | Sample 39 | 35.8 | 2.010 |
| SMA patient | Sample 40 | 18.2 | 2.091 |
| SMA patient | Sample 41 | 249.6 | 1.889 |
| SMA patient | Sample 42 | 212.8 | 1.887 |
| SMA patient | Sample 43 | 424.4 | 1.891 |
| SMA patient | Sample 44 | 9.1 | 2.547 |
| SMA patient | Sample 45 | 31.1 | 1.874 |
| SMA patient | Sample 46 | 22.9 | 2.098 |
| SMA patient | Sample 47 | 313 | 1.897 |
| SMA patient | Sample 48 | 38.0 | 2.051 |
| Healthy control | Sample 1 | 789 | 1.888 |
| Healthy control | Sample 2 | 436 | 2.000 |
| Healthy control | Sample 3 | 1861 | 1.758 |
| Healthy control | Sample 4 | 776 | 1.804 |
| Healthy control | Sample 5 | 436 | 2.250 |
| Healthy control | Sample 6 | 906 | 1.769 |
| Healthy control | Sample 7 | 436 | 1.849 |
| Healthy control | Sample 8 | 559 | 2.009 |
| Healthy control | Sample 9 | 592 | 2.277 |
| Healthy control | Sample 10 | 721 | 1.810 |
| Healthy control | Sample 11 | 565 | 1.954 |
| Healthy control | Sample 12 | 356 | 1.835 |
| Healthy control | Sample 13 | 906 | 1.795 |
| Healthy control | Sample 14 | 756 | 1.821 |
| Healthy control | Sample 15 | 438 | 1.854 |
| Healthy control | Sample 16 | 591 | 1.964 |
| Healthy control | Sample 17 | 600 | 1.899 |
| Healthy control | Sample 18 | 816 | 2.179 |
| Healthy control | Sample 19 | 393 | 1.823 |
| Healthy control | Sample 20 | 762 | 1.856 |
| Healthy control | Sample 21 | 635 | 2.197 |
| Healthy control | Sample 22 | 541 | 1.719 |
| Healthy control | Sample 23 | 640 | 1.817 |
| Healthy control | Sample 24 | 1006 | 1.932 |
| Healthy control | Sample 25 | 219 | 1.823 |
| Healthy control | Sample 26 | 695 | 1.878 |
| Healthy control | Sample 27 | 649 | 2.021 |
| Healthy control | Sample 28 | 515 | 1.743 |
| Healthy control | Sample 29 | 690 | 1.719 |
| Healthy control | Sample 30 | 736 | 1.906 |
| Healthy control | Sample 31 | 702 | 1.857 |
| Healthy control | Sample 32 | 473 | 1.951 |
| Healthy control | Sample 33 | 842 | 1.921 |
| Healthy control | Sample 34 | 493 | 1.978 |
| Healthy control | Sample 35 | 675 | 1.711 |
| Healthy control | Sample 36 | 512 | 1.853 |
| Healthy control | Sample 37 | 758 | 2.082 |
| Healthy control | Sample 38 | 691 | 1.917 |
| Healthy control | Sample 39 | 306 | 1.813 |
| Healthy control | Sample 40 | 730 | 2.080 |
| Healthy control | Sample 41 | 1010 | 1.901 |
| Healthy control | Sample 42 | 673 | 1.866 |
| Healthy control | Sample 43 | 556 | 2.164 |
| Healthy control | Sample 44 | 976 | 1.878 |
| Healthy control | Sample 45 | 1553 | 1.831 |
| Healthy control | Sample 46 | 470 | 1.822 |
| Healthy control | Sample 47 | 730 | 1.966 |
| Healthy control | Sample 48 | 839 | 1.921 |
| Healthy control | Sample 49 | 348 | 1.976 |
| Healthy control | Sample 50 | 533 | 1.817 |
| Healthy control | Sample 51 | 1114 | 1.912 |
| Healthy control | Sample 52 | 548 | 2.347 |
| Healthy control | Sample 53 | 676 | 2.030 |
| Healthy control | Sample 54 | 954 | 1.794 |
| Healthy control | Sample 55 | 431 | 1.883 |
| Healthy control | Sample 56 | 904 | 1.897 |
| Healthy control | Sample 57 | 436 | 2.368 |
| Healthy control | Sample 58 | 810 | 2.027 |
| Healthy control | Sample 59 | 755 | 1.718 |
| Healthy control | Sample 60 | 328 | 1.710 |
| Healthy control | Sample 61 | 1000 | 2.043 |
| Healthy control | Sample 62 | 430 | 1.790 |
| Healthy control | Sample 63 | 600 | 2.220 |
| Healthy control | Sample 64 | 1018 | 2.263 |
| Healthy control | Sample 65 | 411 | 1.818 |
| Healthy control | Sample 66 | 341 | 1.718 |
| Healthy control | Sample 67 | 553 | 1.773 |
| Healthy control | Sample 68 | 417 | 1.781 |
| Healthy control | Sample 69 | 608 | 2.279 |
| Healthy control | Sample 70 | 612 | 2.008 |
| Healthy control | Sample 71 | 455 | 1.953 |
| Healthy control | Sample 72 | 387 | 1.952 |
| Healthy control | Sample 73 | 815 | 1.846 |
| Healthy control | Sample 74 | 308 | 1.820 |
| Healthy control | Sample 75 | 402 | 1.853 |
| Healthy control | Sample 76 | 266 | 1.906 |
| Healthy control | Sample 77 | 563 | 1.956 |
| Healthy control | Sample 78 | 440 | 1.983 |
| Healthy control | Sample 79 | 341 | 2.037 |
| Healthy control | Sample 80 | 823 | 1.714 |
| Healthy control | Sample 81 | 527 | 1.812 |
| Healthy control | Sample 82 | 868 | 1.848 |
| Healthy control | Sample 83 | 772 | 1.815 |
| Healthy control | Sample 84 | 541 | 2.038 |
| Healthy control | Sample 85 | 347 | 1.830 |
| Healthy control | Sample 86 | 418 | 2.035 |
| Healthy control | Sample 87 | 327 | 1.705 |
| Healthy control | Sample 88 | 317 | 1.821 |
| Healthy control | Sample 89 | 496 | 1.706 |
| Healthy control | Sample 90 | 281 | 2.008 |
| Healthy control | Sample 91 | 564 | 1.990 |
| Healthy control | Sample 92 | 359 | 2.022 |
| Healthy control | Sample 93 | 279 | 1.602 |
| Healthy control | Sample 94 | 479 | 2.310 |
| Healthy control | Sample 95 | 522 | 2.187 |
| Healthy control | Sample 96 | 316 | 2.135 |
| Healthy control | Sample 97 | 354 | 2.039 |
| Healthy control | Sample 98 | 758 | 1.919 |
| Healthy control | Sample 99 | 761 | 2.060 |
| Healthy control | Sample 100 | 583 | 1.770 |
| Healthy control | Sample 101 | 804 | 1.831 |
| Healthy control | Sample 102 | 768 | 1.760 |
| Healthy control | Sample 103 | 833 | 2.094 |
| Healthy control | Sample 104 | 652 | 2.218 |
| Healthy control | Sample 105 | 704 | 1.838 |
| Healthy control | Sample 106 | 871 | 3.278 |
| Healthy control | Sample 107 | 670 | 2.000 |
| Healthy control | Sample 108 | 800 | 1.700 |
| Healthy control | Sample 109 | 713 | 2.000 |
| Healthy control | Sample 110 | 279 | 1.963 |
| Healthy control | Sample 111 | 128 | 2.556 |
| Healthy control | Sample 112 | 135 | 2.026 |
| Healthy control | Sample 113 | 255 | 1.891 |
| Healthy control | Sample 114 | 386 | 1.879 |
| Healthy control | Sample 115 | 164 | 1.881 |
| Healthy control | Sample 116 | 334 | 1.961 |
| Healthy control | Sample 117 | 373 | 1.289 |
| Healthy control | Sample 118 | 290 | 1.849 |
| Healthy control | Sample 119 | 265 | 1.636 |
| Healthy control | Sample 120 | 270 | 1.913 |
| Healthy control | Sample 121 | 316 | 1.986 |
| Healthy control | Sample 122 | 384 | 1.826 |
|  |  |  |  |
| Healthy control | Sample 123 | 293 | 1.773 |
| Healthy control | Sample 124 | 123 | 2.133 |
| Healthy control | Sample 125 | 327 | 1.866 |
| Healthy control | Sample 126 | 92 | 1.824 |
| Healthy control | Sample 127 | 222 | 1.932 |
| Healthy control | Sample 128 | 349 | 1.803 |
| Healthy control | Sample 129 | 319 | 1.890 |
| Healthy control | Sample 130 | 216 | 1.835 |
| Healthy control | Sample 131 | 352 | 2.254 |
| Healthy control | Sample 132 | 430 | 1.784 |
| Healthy control | Sample 133 | 536 | 2.152 |
| Healthy control | Sample 134 | 293 | 1.823 |
| Healthy control | Sample 135 | 339 | 1.611 |
| Healthy control | Sample 136 | 262 | 1.804 |
| Healthy control | Sample 137 | 317 | 2.125 |
| Healthy control | Sample 138 | 341 | 1.820 |
| Healthy control | Sample 139 | 331 | 1.708 |
| Healthy control | Sample 140 | 327 | 2.160 |
| Healthy control | Sample 141 | 323 | 2.011 |
| Healthy control | Sample 142 | 533 | 1.962 |
| Healthy control | Sample 143 | 287 | 1.808 |
| Healthy control | Sample 144 | 330 | 2.105 |
| Healthy control | Sample 145 | 421 | 1.813 |
| Healthy control | Sample 146 | 405 | 1.644 |
| Healthy control | Sample 147 | 406 | 2.229 |
| Healthy control | Sample 148 | 309 | 1.855 |
| Healthy control | Sample 149 | 401 | 2.424 |
| Healthy control | Sample 150 | 337 | 2.082 |
| Healthy control | Sample 151 | 583 | 1.899 |
| Healthy control | Sample 152 | 214 | 1.820 |
| Healthy control | Sample 153 | 497 | 2.143 |
| Healthy control | Sample 154 | 312 | 1.830 |
| Healthy control | Sample 155 | 465 | 1.719 |
| Healthy control | Sample 156 | 208 | 1.791 |
| Healthy control | Sample 157 | 505 | 2.168 |
| Healthy control | Sample 158 | 477 | 1.865 |
| Healthy control | Sample 159 | 365 | 2.080 |
| Healthy control | Sample 160 | 126 | 1.750 |
| Healthy control | Sample 161 | 249 | 2.333 |
| Healthy control | Sample 162 | 370 | 2.052 |
| Healthy control | Sample 163 | 661 | 1.902 |
